# POLR3-Related Leukodystrophy: Exploring Potential Therapeutic Approaches

**DOI:** 10.3389/fncel.2020.631802

**Published:** 2021-01-28

**Authors:** Stefanie Perrier, Mackenzie A. Michell-Robinson, Geneviève Bernard

**Affiliations:** ^1^Department of Neurology and Neurosurgery, McGill University, Montréal, QC, Canada; ^2^Child Health and Human Development Program, Research Institute of the McGill University Health Centre, Montréal, QC, Canada; ^3^Department of Pediatrics, McGill University, Montréal, QC, Canada; ^4^Department of Human Genetics, McGill University, Montréal, QC, Canada; ^5^Department of Specialized Medicine, Division of Medical Genetics, Montréal Children’s Hospital and McGill University Health Centre, Montréal, QC, Canada

**Keywords:** POLR3-related leukodystrophy, 4H leukodystrophy, hypomyelination, gene therapy, gene editing, cell therapy

## Abstract

Leukodystrophies are a class of rare inherited central nervous system (CNS) disorders that affect the white matter of the brain, typically leading to progressive neurodegeneration and early death. Hypomyelinating leukodystrophies are characterized by the abnormal formation of the myelin sheath during development. POLR3-related or 4H (hypomyelination, hypodontia, and hypogonadotropic hypogonadism) leukodystrophy is one of the most common types of hypomyelinating leukodystrophy for which no curative treatment or disease-modifying therapy is available. This review aims to describe potential therapies that could be further studied for effectiveness in pre-clinical studies, for an eventual translation to the clinic to treat the neurological manifestations associated with POLR3-related leukodystrophy. Here, we discuss the therapeutic approaches that have shown promise in other leukodystrophies, as well as other genetic diseases, and consider their use in treating POLR3-related leukodystrophy. More specifically, we explore the approaches of using stem cell transplantation, gene replacement therapy, and gene editing as potential treatment options, and discuss their possible benefits and limitations as future therapeutic directions.

## Introduction

Leukodystrophies are a class of heterogeneous inherited neurological diseases characterized by the predominant impairment of the central nervous system (CNS) white matter, with specific involvement of glial cells (Vanderver et al., 2015; Van Der Knaap and Bugiani, 2017). Affected patients typically present in childhood or adolescent years with psychomotor regression and/or neuropsychiatric manifestations. Magnetic resonance imaging (MRI) patterns, followed by genetic investigations, are used to confirm diagnoses (Parikh et al., 2015).

Most leukodystrophies run a progressive disease course, with slow to rapid deterioration after onset, ultimately leading to an early death. Collectively, leukodystrophies affect approximately one in 7,500 individuals, however, there are many different subtypes with varying individual incidence rates (Bonkowsky et al., 2010; Parikh et al., 2015; Adang et al., 2017). Next-generation sequencing has proven to be a valuable first-line diagnostic tool for determining the genetic basis of the disease, and has facilitated the discovery of a variety of causal genes encoding proteins with diverse biological functions (Boycott et al., 2014; Srivastava et al., 2014; Vanderver et al., 2016). Although some leukodystrophies have successful restorative treatments if started early following diagnosis [i.e., pre-or early symptomatic stages (Krivit et al., 1999; Krivit, 2004; Van Den Broek et al., 2018)], most treatments address specific clinical features, providing supportive care (Adang et al., 2017).

Hypomyelinating leukodystrophies (HLDs) are a defined subcategory of leukodystrophies, characterized by defects in initial myelin production and formation during development (Costello et al., 2009; Pouwels et al., 2014; Wolf et al., 2020). HLDs are diagnosed using MRI patterns, notably involving hyperintensity of the white matter compared to gray matter on T2 weighted imaging, and variable signal (i.e., hyperintensity, hypointensity, or isointensity) of white matter on T1 weighted imaging compared to gray matter structures (Schiffmann and Van Der Knaap, 2009; Steenweg et al., 2010; Barkovich and Deon, 2016). Hypomyelination can be diagnosed in a single MRI in children older than 2 years of age, but not in younger children. Indeed, in children below 2 years, the diagnosis of hypomyelination (vs. myelination delay) requires that myelination does not progress between two MRIs taken 6 months apart, with the second performed after 2 years of age (Schiffmann and Van Der Knaap, 2009; Steenweg et al., 2010; Pouwels et al., 2014). As myelination of most key brain areas is virtually complete by 2 years of age, a lack of progression in myelin development seen at this age will likely result in permanent hypomyelination (Steenweg et al., 2010).

Classically, HLDs were primarily known to be caused by pathogenic variants in genes encoding for proteins directly associated with the development, structure, or integrity of the myelin sheath. For example, the prototypical HLD Pelizaeus-Merzbacher disease results from pathogenic variants in *PLP1*, a gene encoding a structural myelin protein (Garbern, 2007). However, a recently growing class of white matter disorders encompasses those caused by pathogenic variants in proteins that play key roles in transcription and translation. For example, pathogenic variants in several genes encoding for aminoacyl tRNA synthetases (e.g., *DARS1, RARS1, EPRS1*) are known to cause HLDs (Park et al., 2008; Taft et al., 2013; Wolf et al., 2014a; Ognjenović and Simonović, 2018; Mendes et al., 2018).

Within the category of white matter disorders caused by defects in transcription/translation-related genes is POLR3-related hypomyelinating leukodystrophy (POLR3-HLD), which is now considered one of the most common HLDs (Schmidt et al., 2020). POLR3-HLD is caused by biallelic pathogenic variants in genes encoding subunits of the transcription complex RNA polymerase III (POLR3), namely *POLR3A, POLR3B, POLR1C*, and *POLR3K* (Bernard et al., 2011; Tétreault et al., 2011; Daoud et al., 2013; Thiffault et al., 2015; Dorboz et al., 2018). POLR3 is responsible for the transcription of several non-coding RNAs (nc-RNAs) which have significant roles in translation and gene expression programs, including transfer RNAs (tRNAs), 5S ribosomal RNA, 7SL and 7SK RNAs, some microRNAs, vault RNAs, and a variety of small nucleolar RNAs, including U6 snRNA (Dieci et al., 2007, 2013; White, 2011; Wu et al., 2012; Lesniewska and Boguta, 2017). As the genes associated with POLR3-HLD have been discovered relatively recently and attempts at generating an animal model were predominantly unsuccessful (Choquet et al., 2017, 2019b), the cellular and molecular mechanisms underlying the white matter pathology of this disease are largely unknown. Research is ongoing regarding the investigation of the pathophysiology of POLR3-HLD; recent modeling of the disease has been accomplished in yeast (Moir et al., 2020), as well as in a conditional mouse model (pre-print data, not yet peer-reviewed; Merheb et al., 2020). Moreover, a variety of different types of pathogenic variants are known to cause POLR3-HLD, including nonsense, missense, intronic, synonymous, and splice site variants, as well as large exonic deletions, and small insertions or deletions (Bernard et al., 2011; Tétreault et al., 2011; Potic et al., 2012; Terao et al., 2012; Daoud et al., 2013; Takanashi et al., 2014; Wolf et al., 2014b; Gutierrez et al., 2015; Thiffault et al., 2015; La Piana et al., 2016; Jurkiewicz et al., 2017; Richards et al., 2017; Al Yazidi et al., 2019; Gauquelin et al., 2019; Harting et al., 2020; Hiraide et al., 2020b; Perrier et al., 2020). It is hypothesized that loss of POLR3 function disrupts the transcription of tRNAs, thereby resulting in dysregulation of global translation during peak periods of myelin development which require synthesis of large amounts of proteins (Pfeiffer et al., 1993; Elbaz and Popko, 2019). An alternative hypothesis involves hypofunction of POLR3 causing impairments in the production of specific nc-RNAs required for the formation of myelin (Choquet et al., 2019a).

**Figure 1:**
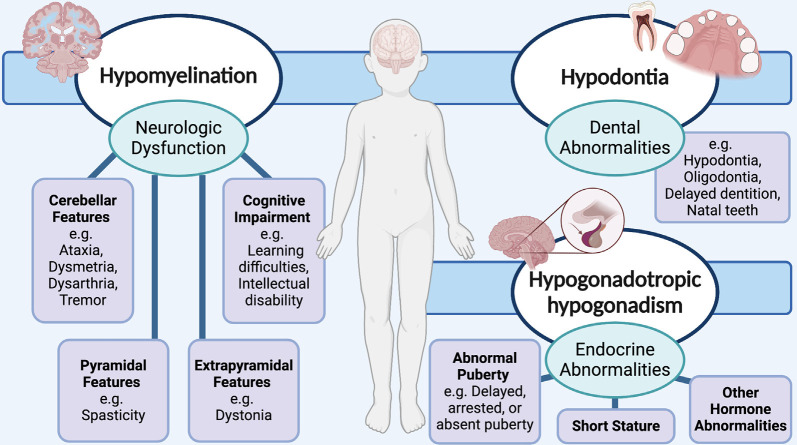
Schematic showing the neurological and non-neurological clinical features that are associated with RNA polymerase III (POLR3)-related, or 4H (hypomyelination, hypodontia, hypogonadotropic hypogonadism) leukodystrophy. Neurological abnormalities typically include cerebellar, pyramidal, extrapyramidal, and cognitive features. Teeth and endocrine abnormalities are also common.

Due to the classic phenotypic presentation of patients involving hypomyelination, hypodontia, and hypogonadotropic hypogonadism, POLR3-HLD is also referred to as 4H leukodystrophy. Before the discovery of the causal genes for POLR3-HLD, four other disorders with a similar set of clinical and MRI features were previously described: ataxia, delayed dentition, and hypomyelination (ADDH; Wolf et al., 2007; Wolff et al., 2010); tremor-ataxia with central hypomyelination (TACH; Bernard et al., 2010, 2011; Tétreault et al., 2011; Tetreault et al., 2012); leukodystrophy with oligodontia (LO; Atrouni et al., 2003; Chouery et al., 2011); and hypomyelination with cerebellar atrophy and hypoplasia of the corpus callosum (HCAHC; Sasaki et al., 2009; Saitsu et al., 2011; Bernard and Vanderver, 2017). Moreover, the clinical phenotype of patients with POLR3-HLD has been extensively characterized *via* large cohort studies, with the most notable features stemming from neurological dysfunction due to hypomyelination (Wolf et al., 2014b; Gauquelin et al., 2019). The typical MRI pattern associated with POLR3-HLD involves diffuse hypomyelination with relative preservation (T2-weighted hypointensity) of the dentate nuclei, anterolateral nuclei of the thalami, globi pallidi, pyramidal tracts at the level of the posterior limb of the internal capsules, and the optic radiations (La Piana et al., 2014; Vrij-Van Den Bos et al., 2017). Thinning of the corpus callosum and cerebellar atrophy have also been noted in a proportion of cases (La Piana et al., 2014; Vrij-Van Den Bos et al., 2017). Neurological features resulting from hypomyelination typically manifest as developmental delay and motor impairment from progressive cerebellar features, such as gait ataxia, tremor, dysmetria, and dysarthria (Bernard and Vanderver, 2017; Figure 1). Extrapyramidal features, most commonly dystonia, have also been reported (Osterman et al., 2012; Al Yazidi et al., 2019), along with pyramidal features such as spasticity, and cognitive features such as learning difficulties and intellectual disability (Wolf et al., 2014b; Bernard and Vanderver, 2017; Gauquelin et al., 2019). Non-neurological features typically involve myopia, abnormal dentition, and endocrine abnormalities including hypogonadotropic hypogonadism, associated with arrested, delayed, or absent puberty, and short stature (Wolf et al., 2014b; Pelletier et al., 2020; Figure 1).

Recently, the spectrum of severity and the associated clinical and MRI features of POLR3-HLD expanded significantly, from very mild to extremely severe. Very mild presentations include asymptomatic young adults, or patients with intellectual disability and milder hypomyelination on MRI compared to the typical phenotype, discovered incidentally during unrelated investigations (Wolf et al., 2014b; Degasperis et al., 2020; Perrier et al., 2020). Isolated hypogonadotropic hypogonadism without evidence of hypomyelination has also been described on the mild end of the POLR3-HLD spectrum (Richards et al., 2017). Patients with the severe form of POLR3-HLD present much earlier compared to the typical phenotype, exhibiting developmental regression, failure to thrive, and severe dysphagia in the first few months of life, with some passing in early childhood due to respiratory complications (Wu et al., 2019; Harting et al., 2020; Perrier et al., 2020). These patients also present with a unique MRI phenotype, in fact not meeting the criteria for hypomyelination, but primarily showing neuronal involvement in specific brain regions (predominantly the putamen and thalamus) with some evidence of insufficient myelin deposition (Perrier et al., 2020). It is hypothesized that the neuronal presentation is likely linked to a specific splicing variant in *POLR3A*, given the common neuronal phenotype shared with other patients harboring the same, or an adjacent, splicing variant in a homozygous or compound heterozygous state (Azmanov et al., 2016; Minnerop et al., 2017; Wu et al., 2019; Harting et al., 2020; Hiraide et al., 2020a). In sum, it is clear that the pathophysiology underlying POLR3-HLD is complex as a broad range of phenotypes are associated with hypomorphic POLR3. In this review article, we will focus on the potential therapeutic options for the classic and most common phenotype, specifically concentrating on hypomyelination.

## POLR3-Related Leukodystrophy: Approaching Treatment Options

With the advent of MRI pattern recognition and improvements in genetic technologies in the last decade, diagnostic rates for leukodystrophies, including POLR3-HLD, have risen in parallel. An important goal for POLR3-HLD research now lies in the determination of quantifiable markers of disease progression. Indeed, before therapeutic options can be considered, clinical outcome measures and surrogate markers of disease progression must be established and deemed accurately quantifiable. These markers are critical for assessing the effectiveness of treatment efficacy in future clinical trials. Advanced neuroimaging techniques, such as diffusion tensor imaging (DTI), pose an interesting route for measurement of improvements in myelination (Aung et al., 2013; Pouwels et al., 2014; Koob et al., 2016; Poretti et al., 2016; Sarret et al., 2018; Van Rappard et al., 2018). The heterogeneity of POLR3-HLD presents an additional limitation for assessing the effectiveness of different therapies as difficulties could arise when comparing the progression rate of phenotypes between patients. Thus far, the clinical experience of most patients with POLR3-HLD presents a relatively similar disease course according to the gene which is mutated. Indeed, those with pathogenic variants in *POLR1C* present with the most severe disease course, followed by *POLR3A*, and then *POLR3B* (Wolf et al., 2014b; Gauquelin et al., 2019). The comparative severity of patients with pathogenic variants in *POLR3K* cannot yet be determined as clinical information has only been published on two patients (Dorboz et al., 2018). In recent years, it has become clear that natural history studies concerning the delineation of disease progression and identification of surrogate markers are of the utmost importance (Pouwels et al., 2014). Hence, it is essential to complete these studies in parallel to pathophysiological investigations for clinical trials of potential therapies to progress.

**Figure 2:**
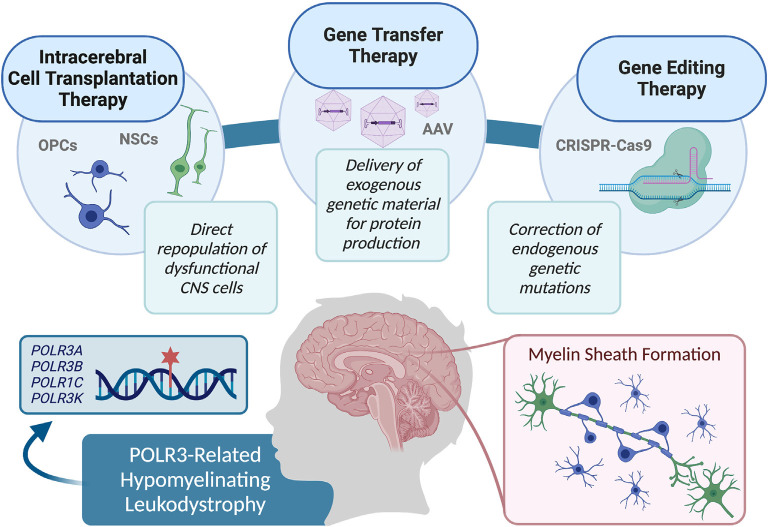
Summary of the therapy approaches that could be explored for use in pre-clinical studies, and eventually translated in clinical trials to treat RNA polymerase III (POLR3)-related leukodystrophy, including cell transplantation therapy, gene transfer therapy, and gene editing techniques.

Limited knowledge of the exact pathophysiological mechanisms underlying POLR3-HLD also poses a challenge for the evaluation of the most effective treatment options. When specific mechanisms are implicated in genetic diseases, it is possible to focus on targeting alternative pathways in treatment approaches, in order to overpass the mechanism containing the defective protein (Greene and Voight, 2016). Although the cellular pathophysiological mechanisms associated with POLR3-HLD have yet to be uncovered, studies have shown that mutations in POLR3 subunits can cause disruptions on several molecular levels. For example, mutational mapping onto specific protein domains suggests association with specific mechanisms of dysfunction, including modification of the catalytic cleft structure, impaired POLR3 complex assembly, perturbed interactions between subunits, and interference within POLR3 complex binding to DNA (Bernard et al., 2011; Tétreault et al., 2011; Girbig et al., 2020; Ramsay et al., 2020). Additionally, protein localization studies have shown that disease-causing *POLR1C* variants can alter assembly and nuclear import exclusively of POLR3, resulting in a lack in binding to POLR3 target genes (Thiffault et al., 2015). Protein expression studies on patient fibroblasts and brain tissue also demonstrate a decrease in POLR3A abundance (Bernard et al., 2011). Finally, functional studies of *POLR3A* mutations associated with POLR3-HLD have demonstrated transcriptional defects when introduced in both yeast and human cells (Choquet et al., 2019a; Moir et al., 2020). Further research on molecular pathways and other POLR3 interactors will be valuable in determining whether suppression of upstream POLR3 inhibitors, such as MAF1 (Reina et al., 2006; Johnson et al., 2007; Bonhoure et al., 2020; Vorländer et al., 2020), are appropriate for future treatments. It is also possible that a small molecule screening approach could identify drugs for the treatment of specific molecular mechanisms, such as upregulation of complex assembly cofactors or signaling molecules for nuclear import of POLR3 (Cloutier and Coulombe, 2010; Lesniewska and Boguta, 2017; Willis and Moir, 2018). However, further research is required in this avenue before a molecular target approach can be considered for the repair of myelin in POLR3-HLD. Currently, as the pathophysiological processes underlying POLR3-HLD are not well known, potential therapeutic approaches can be considered in a general manner, by focusing on the replacement of defective cells (i.e., oligodendrocytes), or directly restoring POLR3 function. Specifically, this could be accomplished by: directly transplanting stem cells containing functional protein to migrate and replicate in damaged areas, using gene therapy techniques to deliver gene products and restore the functional protein in damaged cells, or repairing genetic variants in damaged cells *via* delivery of gene editing constructs (Helman et al., 2015; Gordon-Lipkin and Fatemi, 2018; Figure 2). This review discusses these strategies as potential avenues for treatment of POLR3-HLD, specifically considering its cellular neuropathology and discussing the benefits and limitations of treatments that have been successful in other leukodystrophies, as well as those currently being developed for clinical trials in other genetic diseases.

### Myelination and POLR3-Related Leukodystrophy Cellular Pathology

Myelination is a dynamic process, involving many signaling cues, proteins, and enzymes, that begins *in utero*. The formation of myelin begins in the CNS with the development and migration of oligodendrocyte progenitor cells (OPCs), which extend their processes to contact neuronal axons and begin ensheathment (Michalski and Kothary, 2015). Upon initial axon-glial contact, key myelin membrane components are synthesized and transported to begin the extension of the processes around the axon (Emery, 2010; Mitew et al., 2014). As the processes wrap the axons and begin to compact into several thin layers, the OPCs develop into mature oligodendrocytes, thereby forming the myelin sheath (Baron and Hoekstra, 2010). Compacted myelin allows for rapid propagation of action potentials between neurons, while also providing structural protection to axons. Additionally, complex networks of microtubules in the myelin membrane support the high metabolic demand of the axon by facilitating the transport of proteins, metabolites, and other molecules (Roth et al., 2006; Lee et al., 2012). Typically, myelin deposition begins during the 4th month of gestation *in utero*, and myelination of most major tracts is essentially complete by 2 years of age (Dietrich et al., 1988; Van Der Knaap and Valk, 2005). Myelination continues on a smaller scale into the first and second decades of life, with an increase of approximately 12% in total white matter volume to age 22 (Giedd et al., 1999). Additional changes in white matter volume into adulthood are both regionally and temporally associated with cognitive development and synaptic plasticity, and are also likely associated with axonal factors including pruning, branching, and packing (Sampaio-Baptista and Johansen-Berg, 2017).

Neuropathological investigations of the typical phenotype of POLR3-HLD suggest a complex pathologic process, however, the most prominent feature remains insufficient myelin deposition. The two published cases of typical POLR3-HLD pathology revealed a marked loss of oligodendrocytes, with severity varying in different brain regions (Vanderver et al., 2013; Wolf et al., 2014b). Moderate axonal loss was evident, thought to be secondary to white matter abnormalities due to its apparent proportionality to lack of myelin. Despite the uniform hypomyelinating pattern seen on MRI, it has been hypothesized that POLR3-HLD is a complex heterogenous leukodystrophy with prominent neuroaxonal and glial involvement (Vanderver et al., 2013).

As the neurological manifestations of POLR3-HLD are likely a direct result of the lack of myelin, strategies focusing on restoration of myelin are ideal therapeutic considerations. Moreover, this could be accomplished by replacement of myelinating oligodendrocytes *via* direct delivery of progenitor cells, or by gene therapy aiming at restoring the missing protein in endogenous oligodendrocytes and their progenitors. However, as the neuropathological mechanisms underlying hypomyelination in POLR3-HLD are still undetermined, it is difficult to conclude which option would be most successful in phenotypic remediation. To expand, it is currently unknown how and why cells of the oligodendrocyte lineage in POLR3-HLD are dysfunctional, and whether a defect in proliferation, migration, differentiation, signaling, or the production of myelin *per se* causes hypomyelination. Additionally, it is possible that a combination of cell types may be involved in the disease pathogenesis of POLR3-HLD, and therapies involving the direct target of glial lineages will only be partially effective. Given that POLR3 is ubiquitously expressed, it is conceivable that hypofunction of the protein in cell types other than those that directly produce myelin could play a role in disease pathogenesis. As the white matter of the brain has a complex composition involving the lipid-rich neuron-wrapping myelin sheath composed of oligodendrocytes, as well as astrocytes and microglia that provide structural and trophic support, it can be difficult to focus on specific strategies for its repair without concrete knowledge of the cellular pathogenesis of POLR3-HLD. Gathering more insight into the developmental role of POLR3 in myelin-producing cells and other neural cells, in addition to pathophysiological mechanisms of mutant POLR3 subunits, will allow the field to advance therapeutic strategies based on a deeper understanding of the underlying biology. It is important to note that each therapeutic approach has unique potential benefits and limitations, with the stage of disease progression and patient age remaining strong factors in considering the potential for therapeutic efficacy (Helman et al., 2015; Gordon-Lipkin and Fatemi, 2018).

## Cell-Based Therapies: Transplantation as Treatment for Leukodystrophies

Cellular therapies, which involve the transplantation of stem cells into an affected individual, offer an attractive approach for treating HLDs. Stem cells can self-renew and differentiate into different lineages, including OPCs, and therefore could directly repopulate lost host cells for the regeneration of myelin in leukodystrophies. Generally, the therapeutic mechanisms of cellular therapy can be two-fold, including direct replacement of lost host cells *via* migration of transplanted cells to repopulate defective tissues, and transplanted cells acting as a source of functional exogenous enzymes (De Feo et al., 2012). While delivery of stem cells for the treatment of neurological diseases has been achieved *via* both intravenous and intracerebral administration techniques, only the latter could be applicable for the treatment of POLR3-HLD. Intravenous stem cell therapy, including bone marrow transplantation or hematopoietic stem cell transplantation, has been used in treating other monogenic neurological diseases based on the notion that monocytes could migrate through the blood-brain barrier to the CNS tissue and secrete active enzyme for cellular uptake by dysfunctional host cells, as well as differentiate into microglia and/or astrocytes that could inherently provide trophic support for diseased cells or regulate inflammation (Krivit et al., 1995; Priller et al., 2001; Asheuer et al., 2004; Sun and Kurtzberg, 2018). While this approach has been used in leukodystrophies that are associated with enzyme deficiencies [e.g., globoid cell leukodystrophy or Krabbe disease (Escolar et al., 2005; Wright et al., 2017; Laule et al., 2018), adrenoleukodystrophy (Peters et al., 2004; Mahmood et al., 2007; Matsukawa et al., 2020), and metachromatic leukodystrophy (Martin et al., 2013; Musolino et al., 2014; Boucher et al., 2015; Groeschel et al., 2016)], it is not applicable for treatment of the hypomyelinating phenotype associated with POLR3-HLD as neither the POLR3 enzyme complex nor its subunits are secreted extracellularly for reuptake, and myelination would be dependent on the delivery of functional OPCs or earlier lineages. Therefore, intracerebral administration of stem cells of neural lineage poses the most likely route for exploration in the treatment of POLR3-HLD.

### Neural Stem Cell Transplantation and Remyelination

Neural stem cells (NSCs) are multipotent neural cells that give rise to radial glial progenitor cells, which can, in turn, give rise to neuron and glial cell populations, making them an attractive cell type for transplantation in the leukodystrophy setting (Brüstle et al., 1997; Temple, 2001; Zhao and Moore, 2018). During neural development, gradients of specific signaling molecules guide the fate of NSCs and provide positional information to form different regions of the brain (Wolpert, 1994; Temple, 2001). In the CNS, NSCs also have a temporal differentiation component, where the response to growth factors is altered over time, as the cells undergo repeated asymmetric divisions to first produce neurons, followed by glia (Qian et al., 2000; Okano and Temple, 2009). In the postnatal brain, NSC production and neurogenesis are restricted to certain brain areas but primarily occur in the subventricular zone (Gonzalez-Perez, 2012).

Several mouse models of dysmyelination and hypomyelination have shown that intracerebral-transplanted NSCs are effective in remyelinating the myelin-deficient brain (Duncan et al., 2011). Explored extensively is the *shiverer* mouse model, which exhibits dysmyelination and a motor phenotype due to a deletion in the *Mbp* gene, encoding for myelin basic protein, which is required for the formation of major dense lines in compact myelin (Privat et al., 1979; Roach et al., 1985). When transplanted into *shiverer* mice, NSCs can differentiate and remyelinate the brain, promoting recovery of their ataxic phenotype and prolonging survival (Yandava et al., 1999; Low et al., 2009; Uchida et al., 2012). Additionally, when transplanted into the *shiverer* spinal cord, exogenous transplanted NSCs can ensheath axons, form compact myelin, and improve nerve conduction (Eftekharpour et al., 2007; Mothe and Tator, 2008; Buchet et al., 2011). Studies on rodent models of Pelizaeus-Merzbacher disease have shown similar results; following NSC transplantation, *Plp1*-transgenic mutant mice undergo remyelination of the brain with the production of compact myelin (Marteyn et al., 2016; Gruenenfelder et al., 2020). Additionally, engraftment of NSCs into the white matter tracts of hypomyelinated mutant myelin-associated glycoprotein and nonreceptor-type tyrosine kinase Fyn (MAG/Fyn) mice produced mature oligodendrocytes and improvements in myelination (Ader et al., 2001, 2004). These studies provide evidence that mammalian NSCs can undergo functional integration into the CNS white matter, promoting remyelination and offering potential as a therapeutic approach in hypomyelinating disorders. Besides direct remyelination, it is also thought that NSC transplantation can offer an additional advantage through a neuroprotective effect *via* the release of trophic factors, which promote tissue repair and protect endogenous cells from further damage (De Feo et al., 2012).

Recently, the safety of allogeneic NSC intracerebral transplantation in humans was investigated in a phase I clinical trial including four young patients with Pelizaeus-Merzbacher disease, who were monitored over the course of 5 years (Gupta et al., 2012, 2019). The primary goal of this study was to assess the safety profile of the transplantation of allogeneic NSCs derived from human fetal brain tissue using intracerebral injections. Using MRI guidance, cells were delivered *via* four bilateral frontal burr holes to the deep white matter of the centrum semiovale or corona radiata, and patients underwent an immunosuppression regime. A 1-year evaluation determined that the procedure was well-tolerated without clinical or radiological adverse effects, and after 5-years, no tumor formation was evident and no other long-term adverse effects were noted. However, two patients had an immune response and developed donor-specific leukocyte antigen alloantibodies, pointing to the importance of monitoring immune response in future studies. Serial MRI and magnetic resonance spectroscopy (MRS), including DTI, were performed for evaluation of remyelination, where signal changes were observed at the injection sites and some distant regions in each patient through the second year following transplantation. In the three patients who were studied up to year 5, persistent increased signal changes were noted, however, they were described as patchy and subtle, and could not be guaranteed conclusive evidence of remyelination. Although further studies are required to optimize transplantation efficacy, this study provides support for the safety of intracerebral transplantation of progenitor cells for repopulation of myelin in HLDs.

Should the transplanted NSCs be successful in migrating, signaling, and differentiating to form functional myelin in humans, this therapeutic approach would be optimal to treat the diffuse hypomyelination seen in POLR3-HLD. However, in considering the described rodent studies of remyelination following NSC transplantation, results should be interpreted with caution for their translation to the clinical setting as rodents have a much lower proportion of subcortical white matter in relation to cortical volume compared to humans (Schoenemann et al., 2005; Hofman, 2014). Therefore, transplantation in humans would more heavily depend on the severity of hypomyelination and the extent to which exogenous cells must migrate and reproduce. Moreover, to effectively correct CNS functioning *via* remyelination in humans, experimental studies on higher-order mammals, such as primates, would allow for a more comparable result in terms of determining the optimal dosage and regions of transplantation.

### Glial Progenitor Cell Transplantation: A Targeted Lineage

Glial progenitor cells (GPCs), which are further patterned from NSCs towards a glial fate, have also been explored as a candidate for cerebral transplantation in leukodystrophies (Osorio and Goldman, 2016; Goldman, 2017; Chanoumidou et al., 2020). Similar to NSCs, GPCs can be generated from pluripotent stem cells or harvested and purified from fetal brain tissue for transplantation (Nunes et al., 2003; Monaco et al., 2012). Many studies have successfully performed intracerebral transplantation of glial cells in animal models and shown their effectiveness in remyelination (Duncan, 2005; Franklin and Ffrench-Constant, 2008; Goldman, 2011). Notably, several studies involving the transplantation of human glial lineage-specific cells into *shiverer* mice show consistent results, with evidence of robust remyelination, prolonged survival, and phenotypic rescue (Windrem et al., 2004, 2008, 2014, 2020; Izrael et al., 2007; Mariani et al., 2019). These results provide support for clinical exploration of this treatment, revealing that GPCs have a migratory potential and can effectively differentiate *in vivo* when transplanted into another host. Comparative efficacy between specific lineages in transplantation therapy remains to be confirmed; in one study, both NSCs and OPCs were able to remyelinate and produce compact myelin in both Pelizaeus-Merzbacher disease *Plp1-*overexpressing and *shiverer* immunodeficient mouse models, however, in the transgenic *Plp1-*overexpressing mice, NSCs more notably promoted survival and prolonged lifespan, whereas in *shiverer* mice, OPC transplantation promoted a slightly longer lifespan compared to NSCs (Marteyn et al., 2016). Nonetheless, it is important to note that the microenvironment within the CNS tissue likely had a significant impact on survival, with neuroinflammation being downregulated in NSC-grafted mice, which is an important consideration in therapy for Pelizaeus-Merzbacher disease due to the known inflammatory component of disease pathogenesis (Marteyn et al., 2016). Likewise, when OPCs were co-transplanted with mesenchymal stem cells (MSCs) into *shiverer* mice, the immune response was minimized and increased oligodendrocyte engraftment, myelination, and maturation was evident (Cristofanilli et al., 2011). Therefore, immune response could prove to be an additional important consideration when evaluating the effectiveness of stem cell therapy, and would be noteworthy to explore in POLR3-HLD pathogenesis before the development of therapeutic strategies.

### Induced Pluripotent Stem Cells: Patient-Derived Cell Therapy Approaches

GPCs generated from induced pluripotent stem cells (iPSCs) have also been investigated as a prospect for cell therapy and transplantation in white matter diseases (Fox et al., 2014; Chanoumidou et al., 2020). iPSC-derived cells provide an additional advantage as they harbor the genetic background of the individual from whom they originate, thereby adding a patient-specific approach to cell-based therapies. iPSCs can be generated *via* direct reprogramming of somatic cells using a series of pluripotency factors, reverting them into a stem-like fate with the ability to renew indefinitely or differentiate into the desired lineage (Takahashi and Yamanaka, 2006; Takahashi et al., 2007). Patient-specific cells with a renewable potential are especially appealing for the treatment of genetic disorders as they can be expanded to a large number before transplantation and downstream differentiation, and importantly they can evade the possible immunologic rejection that accompanies allogeneic stem cell transplantation. However, before iPSC-derived GPCs can be considered in a clinical setting, there are several limitations to consider and study, including the possibility of tumor formation, as well as potential safety concerns of gene editing required for correction of disease-causing mutations in patient cells, including off-target effects, immunotoxicity, and DNA damage toxicity (Neofytou et al., 2015; Uddin et al., 2020).

Studies on the development of iPSC-derived oligodendrocytes have progressed in the past decade, leading to increased discussion of their utility in treating neurological diseases (Chanoumidou et al., 2020). One of the first studies of human iPSC-derived OPCs aimed to investigate their myelinating potential in the lysolecithin-induced demyelinated rat optic chiasm, in which remyelination was evident following transplantation, reinforcing the potential for iPSC-derived cell transplantation (Pouya et al., 2011). Following this direction, further studies were completed transplanting human iPSC-derived cells into the *shiverer* mouse, revealing that iPSC-derived OPCs can migrate and robustly myelinate brain tissue (Sim et al., 2011; Wang et al., 2013; Ehrlich et al., 2017). iPSC-derived cell transplantation has also proven effective in other neurodegenerative disease models; transplantation studies using a mouse model for Huntington’s disease recently demonstrated that iPSC-derived NSCs were capable of ameliorating their motor phenotype and differentiating into region-specific neurons without tumor formation, thereby providing the foundation for use of iPSC-derived cells in future studies of neurological diseases (Al-Gharaibeh et al., 2017).

Using a direct approach to replace myelin in the brain *via* NSCs, GPCs, or OPCs is an option to consider further studying in POLR3-HLD, however, studies are needed to first determine the pathophysiological mechanisms underlying hypomyelination. The use of autologous patient-specific iPSCs is also an attractive approach due to the decreased risk of transplant rejection. In these circumstances, the concern for donor cell rejection would be limited given that the patient-derived cells are nonimmunogenic, and therefore suppression of the immune system could be avoided. Moreover, iPSCs offer an accessible and renewable source of patient-derived cells, making them an optimal option for transplantation, provided that the potential for tumor formation is deemed very low risk. Further research into the potential for genetic correction of iPSCs from POLR3-HLD patients would be required to determine whether restoration of myelin would be possible with iPSC-derived OPC transplantation.

## Gene Transfer Therapy: Considerations in Leukodystrophies

Historically, the concept of gene therapy evolved from gene transfer experiments which suggested that supplying functional transgenes to cells with corresponding dysfunctional counterparts might provide therapeutic benefit (Rogers, 1959, 1966, 1971; Rogers and Pfuderer, 1968; Terheggen et al., 1975; Friedmann, 2001; Wirth et al., 2013). Gene therapy as a field has grown beyond gene transfer therapy, to encompass techniques such as oligonucleotide and mRNA therapy (Bennett, 2019; Kowalski et al., 2019; Setten et al., 2019) as well as gene editing (discussed below). Typically, gene transfer therapy is divided into *ex vivo* and *in vivo* approaches that make use of different viral vectors for delivery of genetic material to cells. *Ex vivo* gene therapy usually involves removing hematopoietic stem cells from the body and administering a gene therapy vector (often lentiviral) *in vitro* before re-infusing treated cells into the patient. It is methodologically similar to bone marrow transplantation therapy and has overlapping applications, with the advantage of obviating the need for long-term immunosuppression required after bone marrow transplantation. *Ex vivo* gene therapy of this type has been used successfully to treat X-linked adrenoleukodystrophy (Cartier et al., 2009, 2012; Eichler et al., 2017) and metachromatic leukodystrophy (Biffi et al., 2013; Sessa et al., 2016) if administered early. *Ex vivo* gene therapy using hematopoietic stem cells is not a viable option for POLR3-HLD due to the primary defect in POLR3 activity in brain tissues, especially because the complex is not secreted and has a primarily non-metabolic function. However, *ex vivo* gene therapy may have an application using iPSCs, if treated cells are subsequently differentiated to a glial lineage and delivered into the brain. Limitations would involve similar factors to those described above (i.e., safety, route of transplantation, migration, and differentiation capacity). Primarily, *in vivo* gene transfer has been applied successfully to treat specific leukodystrophies, and may be a candidate modality for the treatment of POLR3-HLD. Here, we will focus on *in vivo* gene transfer data, which represents a majority of the literature and clinical experience with gene therapy directly targeting the brain in leukodystrophies, such as Canavan’s disease and metachromatic leukodystrophy. As our knowledge of POLR3-HLD pathology continues to evolve, so too will opportunities to advance tractable strategies for developing a disease-modifying treatment.

### *In vivo* Approaches to Gene Therapy

In considering *in vivo* gene therapy, several viral vectors have been proposed to achieve transgene delivery, but thus far, the most clinically successful has been adeno-associated virus (AAV). *In vivo* gene therapy for leukodystrophies began with an AAV trial for Canavan disease, an autosomal recessive leukodystrophy caused by mutations in the *ASPA* gene, encoding the enzyme aspartoacyclase which functions to degrade N-acetylaspartate (NAA) in the brain (Janson et al., 2002). AAV2-*ASPA* treatment was supported by concurrent pre-clinical rodent studies suggesting human *ASPA* gene transfer to Canavan mice and rats resulted in decreased NAA concentrations in brain tissue, along with decreased seizure frequency and histopathological improvements (Matalon et al., 2003; Mcphee et al., 2005). These findings were translated into a clinical trial. Long term follow-up in a cohort of 28 patients, 13 of which were treated by intraparenchymal delivery of AAV2-*ASPA* to six sites in the brain, demonstrated a good safety profile with the most common adverse events (i.e., small subdural hemorrhage, postoperative fever) most likely associated with the neurosurgical aspect of the treatment, and no adverse events occurring after 90 days of follow-up (Leone et al., 2012). AAV2-*ASPA* was shown to decrease NAA in the brain, as measured by MRS, as well as the slow progression of brain atrophy, and was considered to have been associated with adequate safety and moderate overall clinical efficacy that warranted further clinical trials (Leone et al., 2012). This early AAV trial was instrumental in demonstrating the enhanced safety profile of AAV for *in vivo* gene therapy in leukodystrophies.

The discovery of novel AAV serotypes in nonhuman primates and human tissues elucidated numerous aspects of AAV biology, including their differences in tissue tropism, leading to an explosion of studies exploring the use of naturally occurring AAV serotypes and recombinant AAV (Gao et al., 2002, 2003, 2004). Importantly, the AAV serotypes identified in the course of Dr. Gao and colleagues’ work especially AAV9, have been studied for their utility in transducing brain tissues. An important aspect surrounding the use of AAV9 for CNS diseases involves its enhanced ability to target the CNS, which allows for intrathecal or intravenous administration (Foust et al., 2009; Mendell et al., 2017; Gessler et al., 2019). Most notably, AAV9 was successfully used in a clinical trial for spinal muscular atrophy (Mendell et al., 2017), resulting in FDA approval of Zolgensma^®^, an intravenously delivered gene therapy treatment. AAV9 has also recently demonstrated effectiveness in a mouse model of Canavan disease (Gessler et al., 2017), which played a role in promoting the rAAV9-*ASPA* vector transitioning to a recent open-label clinical trial for Canavan disease (*CANaspire*, ASPA Therapeutics). Finally, an exciting recent AAV9 finding is the success of AAV9-*GALC* in treating a canine model of globoid cell leukodystrophy or Krabbe disease, improving myelination and extending lifespan more than seven times beyond the typical life expectancy for model animals (Bradbury et al., 2020). However, AAV9 is not known to efficiently mediate significant transduction of oligodendrocyte lineage cells.

A Clade E AAV serotype identified in 2004 (Gao et al., 2004) called AAVrh.10, has been tested in the context of metachromatic leukodystrophy on a small number of patients (NCT01801709); however, the results of this trial have not been released. The initial preclinical data for this study suggested that intracerebral delivery of AAVrh.10-*ARSA* was superior to AAV5 both in terms of the overall impact on the model disease and its ability to transduce oligodendrocytes (Sevin et al., 2006, 2007; Piguet et al., 2012), which led to further safety and feasibility assessments in non-human primates leading up to the clinical trial (Zerah et al., 2015). Importantly, in the preclinical assessment, Sevin and colleagues evaluated the direct impact of AAVrh.10 on oligodendrocyte transduction using a GFP-containing vector and estimated that 9% of oligodendrocytes in the striatum were transduced directly, whereas 21% were found to contain ARSA enzyme after administration of AAVrh.10-*ARSA* (Piguet et al., 2012). These findings indicate that cross-correction of oligodendrocyte ARSA enzyme levels *via* transduction of non-oligodendrocyte targets plays a role in the observed improvement in oligodendrocyte sulfatide levels and brain pathology (Piguet et al., 2012). The AAVrh.10 trial excepted, in each of the mentioned leukodystrophies in which *in vivo* gene therapy has been tested, the putative improvement in oligodendrocyte function is thought to occur through cross-correction. Indeed, most AAV capsids are not known to efficiently transduce oligodendrocytes (Burger et al., 2004; Cearley and Wolfe, 2006; Cearley et al., 2008; San Sebastian et al., 2013). This fact has prompted studies evaluating the use of oligodendrocyte-specific promoters to drive expression in oligodendrocytes (Chen et al., 1998; Lawlor et al., 2009) as well as the pursuit of novel recombinant capsids with significant oligodendrocyte tropism as demonstrated in rodents (Powell et al., 2016), and the characterization of oligodendrocyte tropism in a novel naturally occurring AAV capsid (Hsu et al., 2020). Taken together, these study results indicate that AAV vector research continues to yield important advances toward achieving both safety and efficacy for *in vivo* gene therapy approaches to leukodystrophies. The increasing focus on understanding how AAV technology can be used to target oligodendrocyte lineage cells will be important for the development of an *in vivo* gene therapy approach to POLR3-HLD.

Currently, the POLR3-HLD disease population is divided with the majority (≥90%) of patients having either biallelic mutations in *POLR3A* or *POLR3B* (Bernard et al., 2011; Tétreault et al., 2011; Daoud et al., 2013; Wolf et al., 2014b) and a minority (<10%) having mutations in *POLR1C* (Thiffault et al., 2015; Gauquelin et al., 2019) or *POLR3K* (Dorboz et al., 2018). In the future, it may be possible to treat patients by grouping according to the affected subunit and administering a vector carrying the appropriate sequence *in vivo*. However, there are three key challenges for developing an *in vivo* gene therapy approach for POLR3-HLD that have not been addressed by prior *in vivo* leukodystrophy gene therapy studies. The first is the fact that in each of the previously mentioned diseases, cross-correction is possible and beneficial due to the nature of the defective enzymes and metabolites responsible for the disease. In POLR3-HLD, cross-correction is improbable because POLR3 subunits are unlikely to be secreted or transferred between cells and also because pathogenesis likely does not involve the accumulation of the enzymatic reactants (RNA nucleotides), as they are used by other RNA polymerases, and do not directly cause toxicity. Therefore, directly correcting the oligodendrocyte lineage is an important aspect of a putative gene therapy strategy for POLR3-HLD. Second, the pathophysiological axis of POLR3-HLD is hypomyelination, relating to a specific and yet poorly characterized deficit in the oligodendrocyte lineage that may occur well before the cells mature and myelinate the affected CNS regions. If the deficit occurs primarily in the cellular precursors of oligodendrocytes (i.e., a dividing cell population), the exponential dilution of non-integrating vector genomes such as those transduced using AAV is an important consideration. Third, attempts to produce a representative animal model of POLR3-HLD in which to perform pre-clinical testing have proven difficult (Choquet et al., 2017, 2019b), and this barrier will need to be overcome to properly test any novel therapeutic candidate. Recently, progress in generating an animal model has been made using an *Olig2*-Cre conditional double *Polr3a* mutant knock-in strategy, in a recent pre-print which has yet to be peer-reviewed at the time of writing (Merheb et al., 2020). Overcoming these challenges would elucidate the potential for POLR3-HLD gene transfer, and will also inform the future development of more advanced and/or personalized (e.g., gene editing) therapeutic strategies for POLR3-HLD.

## Gene Editing Techniques: A Modern Approach

Recently, gene-editing research has gained traction for its utilization in the development of patient-specific therapies for genetic diseases. While these techniques are not yet employed in a large-scale clinical setting, they hold promise for treating rare genetic diseases that are without curative therapies. Moreover, the design of personalized therapies is a possibility through the use of gene editing, a technique that can create alterations in precise genomic locations to correct pathogenic variants. Yet, to establish translational gene editing strategies, additional aspects must be investigated such as vehicles and delivery methods of editing systems, optimization of editing constructs, and elimination of off-target effects. Furthermore, with correct optimization, genome engineering can lead to the establishment of personalized therapies for diseases that are otherwise challenging to treat.

### CRISPR-Cas9 Editing System

Since the discovery of its potential for human genome editing in 2013, clustered regularly interspaced short palindromic repeats (CRISPR)-Cas gene-editing technology has been heavily investigated for its use in studying and treating genetic diseases (Cong et al., 2013; Jinek et al., 2013). The CRISPR-Cas system harnesses the cellular machinery involved in the adaptive immune response of bacterial cells against viral particles (Horvath and Barrangou, 2010). This highly specific system can target precise genomic regions and has revolutionized modern genetic research for its capability to easily manipulate the human genome. While different systems have been engineered using a series of CRISPR/Cas components and types of Cas nucleases, the Cas9 nuclease has been most commonly used in genetic editing of mammalian cells (Makarova and Koonin, 2015). In combination with a single guide-RNA (sgRNA), Cas9 can be programmed to target and cleave complementary DNA sequences, which can be subsequently repaired using a donor DNA template strand and the intrinsic homology-directed repair mechanism (Mali et al., 2013; Ran et al., 2013; Yang et al., 2014). Thus, given that the proper cell type is targeted, it is possible to use CRISPR-Cas9 technology to genetically correct mutations causing monogenic diseases, bringing to light its ability to facilitate phenotypic repair.

CRISPR-Cas9 editing has been successfully used in many *in vitro* and *in vivo* studies to both explore disease pathophysiology through the creation of transgenic or knock-out models, and investigate treatment methods *via* targeted gene editing and correction of genetic mutations (Rodríguez-Rodríguez et al., 2019). For example, in an initial study using a murine model of hereditary tyrosinemia, injection of CRISPR-Cas9 components into the liver led to phenotypic rescue, demonstrating the potential for genetic correction *in vivo* (Yin et al., 2014). Additionally, studies using the *mdx* murine model for Duchenne muscular dystrophy have shown that delivery of CRISPR-Cas9 constructs, both at the germline level with injection into zygotes and postnatally with delivery *via* AAV9, can lead to phenotypic improvements (Long et al., 2014). *In vitro* and *in vivo* studies have also been completed using CRISPR-Cas9 to correct mutations associated with Huntington’s disease, with promising results demonstrating that suppression of mutant alleles can alleviate motor phenotypes in mice (Shin et al., 2016; Kolli et al., 2017; Monteys et al., 2017; Yang et al., 2017). In the field of HLDs, a recent study has demonstrated that CRISPR-Cas9 mediated germline suppression of *Plp1* in the severe *jimpy* mouse model of Pelizaeus-Merzbacher disease leads to increased myelination and restored lifespan (Elitt et al., 2020). Most recently, the first clinical trials of CRISPR-Cas9 therapies have launched, with *ex vivo* approaches centering around cancer immunotherapy, as well as gene disruption of hematological disorders including sickle-cell anemia and β-thalassemia (Li et al., 2020; Rosenblum et al., 2020; Uddin et al., 2020). Additionally, an *in vivo* approach has been employed in Leber congenital amaurosis, a monogenic disease associated with childhood blindness, involving the delivery of AAV5-packaged CRISPR-Cas9 constructs directly to the retina (Maeder et al., 2019). As these trials progress and with the assessment of long-term outcomes and safety, this gene-editing technology could show powerful potential for use in treating many classes of diseases. Moreover, research involving gene editing with CRISPR-Cas9 techniques in the CNS is constantly evolving; innovations and improvements to the editing system focus on optimizing editing efficiencies and reducing off-target effects, as well as exploring delivery methods *via* biological vesicles, nanoparticles, or viruses (Cota-Coronado et al., 2019;Sandoval et al., 2020).

While rapidly advancing, gene editing techniques would have to be studied *in vitro* and *in vivo* for their use in correcting the POLR3-HLD phenotype before they can be considered as a potential therapeutic approach. It is noteworthy that the use of CRISPR-Cas9 technology is not effective for the correction of all mutation types associated with POLR3-HLD (i.e., large exonic deletions, synonymous variants, some splice site variants), and this therapeutic approach would have to be considered on a patient-specific level. Moreover, this technique is still in the early experimental stage of study and before it can be considered in a clinical setting, its benefits and downfalls as a therapeutic tool must be explored along with the most optimal delivery methods and its potential in correcting cells of the CNS. In speculating on the use of gene editing therapy to treat the cellular pathogenesis associated with POLR3-HLD, this therapy may or may not be applicable depending on the stage of oligodendrocyte lineage that is defective. Moreover, if future studies find that early OPC proliferation or migration ability is not severely affected, and pathogenesis predominantly concerns the formation of myelin itself (due to transcriptional defects causing lack of protein availability for myelin membrane formation), the delivery of CRISPR constructs for genetic correction of myelinating cells could show high potential for phenotypic remediation. However, there are many other potential scenarios in which different cell types or mechanisms could be affected (e.g., differentiation of NSCs to a glial fate, impairments in migration of OPCs, maturation of OPCs into oligodendrocytes, signaling between different cell types and/or other mechanisms for formation, wrapping, or compaction of the myelin membrane itself). Thus, without knowledge on the cellular pathophysiology, it is to be determined whether correcting cells after birth and the initial waves of OPC production/migration during the *in utero* period of myelin development would be applicable. Knowledge of disease pathogenesis would help predict the probability of success for delivery of gene editing constructs at certain stages of the disease progression, and whether myelination is possible.

## Conclusion: The Future of POLR3-HLD Therapies

Along the front of therapy development for rare inherited neurological disorders, advances in cell therapy, gene therapy, and gene editing techniques have all presented exciting results in recent years. Combination approaches have also been considered, including the use of gene transfer or editing of stem cells for transplantation to improve disease phenotypes (Ricca et al., 2015; Meneghini et al., 2017). In considering POLR3-HLD, much information remains to be uncovered regarding the pathophysiology of the disease and whether myelin restoration is possible. As pathological studies demonstrate that oligodendrocytes are primarily affected in POLR3-HLD, this review provided a cell-specific approach to the consideration of therapies. However, disease pathogenesis may involve other cell types, which could also be targeted in combination. The described therapies offer potential options for exploration, and future studies in both cellular and animal models to investigate their effectiveness and mechanisms would prove to be beneficial. Moreover, developing disease biomarkers and tangible clinical outcome measures are of utmost importance to evaluate therapeutic efficacy and successfully translate pre-clinical findings into the clinical setting. Ongoing research on POLR3-HLD pathophysiology will surely provide a backbone for ascertaining which therapy approaches could provide the most beneficial results, and ultimately uncover the avenues for potential clinical trial development to improve patient outcomes.

## Author Contributions

SP, MMR, and GB conceived the design of the article, reviewed the literature, and contributed to the writing and editing of the manuscript. All authors contributed to the article and approved the submitted version.

## Conflict of Interest

The authors declare that the research was conducted in the absence of any commercial or financial relationships that could be construed as a potential conflict of interest.

## Abbreviations

4H: hypomyelination, hypodontia, hypogonadotropic hypogonadism
AAV: adeno-associated virus
CNS: central nervous system
CRISPR: clustered short regularly interspaced palindromic repeats
DTI: diffusion tensor imaging
GPC: glial progenitor cell
HLD: hypomyelinating leukodystrophy
LD: leukodystrophy
MRI: magnetic resonance imaging
MRS: magnetic resonance spectroscopy
NAA: N-acetylaspartate
NSC: neural stem cell
nc-RNAs: non-coding RNAs
OPC: oligodendrocyte progenitor cell
POLR3-HLD: POLR3-related hypomyelinating leukodystrophy
tRNAs: transfer RNAs.

## References

[B1] Adang L. A. Sherbini O. Ball L. Bloom M. Darbari A. Amartino H.. (2017). Revised consensus statement on the preventive and symptomatic care of patients with leukodystrophies. Mol. Genet. Metab. 122, 18–32. 10.1016/j.ymgme.2017.08.006 28863857 PMC8018711

[B2] Ader M. Schachner M. Bartsch U. (2001). Transplantation of neural precursor cells into the dysmyelinated CNS of mutant mice deficient in the myelin-associated glycoprotein and Fyn tyrosine kinase. Eur. J. Neurosci. 14, 561–566. 10.1046/j.0953-816x.2001.01673.x 11553306

[B3] Ader M. Schachner M. Bartsch U. (2004). Integration and differentiation of neural stem cells after transplantation into the dysmyelinated central nervous system of adult mice. Eur. J. Neurosci. 20, 1205–1210. 10.1111/j.1460-9568.2004.03577.x 15341592

[B5] Al-Gharaibeh A. Culver R. Stewart A. N. Srinageshwar B. Spelde K. Frollo L.. (2017). Induced pluripotent stem cell-derived neural stem cell transplantations reduced behavioral deficits and ameliorated neuropathological changes in YAC128 mouse model of Huntington’s disease. Front. Neurosci. 11:628. 10.3389/fnins.2017.00628 29209158 PMC5701605

[B4] Al Yazidi G. Tran L. T. Guerrero K. Vanderver A. Schiffmann R. Wolf N. I.. (2019). Dystonia in RNA polymerase III-related leukodystrophy. Mov. Disord. Clin. Pract. 6, 155–159. 10.1002/mdc3.12715 30838315 PMC6384176

[B6] Asheuer M. Pflumio F. Benhamida S. Dubart-Kupperschmitt A. Fouquet F. Imai Y.. (2004). Human CD34^+^ cells differentiate into microglia and express recombinant therapeutic protein. Proc. Natl. Acad. Sci. U S A 101, 3557–3562. 10.1073/pnas.0306431101 14990803 PMC373501

[B7] Atrouni S. Daraze A. Tamraz J. Cassia A. Caillaud C. Megarbane A. (2003). Leukodystrophy associated with oligodontia in a large inbred family: fortuitous association or new entity? Am. J. Med. Genet. A 118A, 76–81. 10.1002/ajmg.a.10019 12605447

[B8] Aung W. Y. Mar S. Benzinger T. L. (2013). Diffusion tensor MRI as a biomarker in axonal and myelin damage. Imaging Med. 5, 427–440. 10.2217/iim.13.49 24795779 PMC4004089

[B9] Azmanov D. N. Siira S. J. Chamova T. Kaprelyan A. Guergueltcheva V. Shearwood A. J.. (2016). Transcriptome-wide effects of a POLR3A gene mutation in patients with an unusual phenotype of striatal involvement. Hum. Mol. Genet. 25, 4302–4314. 10.1093/hmg/ddw263 27506977

[B10] Barkovich A. J. Deon S. (2016). Hypomyelinating disorders: an MRI approach. Neurobiol. Dis. 87, 50–58. 10.1016/j.nbd.2015.10.015 26477299

[B11] Baron W. Hoekstra D. (2010). On the biogenesis of myelin membranes: sorting, trafficking and cell polarity. FEBS Lett. 584, 1760–1770. 10.1016/j.febslet.2009.10.085 19896485

[B12] Bennett C. F. (2019). Therapeutic antisense oligonucleotides are coming of age. Ann. Rev. Med. 70, 307–321. 10.1146/annurev-med-041217-010829 30691367

[B14] Bernard G. Chouery E. Putorti M. L. Tétreault M. Takanohashi A. Carosso G.. (2011). Mutations of POLR3A encoding a catalytic subunit of RNA polymerase Pol III cause a recessive hypomyelinating leukodystrophy. Am. J. Hum. Genet. 89, 415–423. 10.1016/j.ajhg.2011.07.014 21855841 PMC3169829

[B15] Bernard G. Thiffault I. Tetreault M. Putorti M. L. Bouchard I. Sylvain M.. (2010). Tremor-ataxia with central hypomyelination (TACH) leukodystrophy maps to chromosome 10q22.3-10q23.31. Neurogenetics 11, 457–464. 10.1007/s10048-010-0251-8 20640464 PMC4147760

[B13] Bernard G. Vanderver A. (2017). “POLR3-related leukodystrophy,” in GeneReviews, eds Adam M. P. Ardinger H. H. Pagon R. A. Wallace S. E. Bean L. J. H. Stephens K. (Seattle, WA: University of Washington), 1993–2020. 22855961

[B16] Biffi A. Montini E. Lorioli L. Cesani M. Fumagalli F. Plati T.. (2013). Lentiviral hematopoietic stem cell gene therapy benefits metachromatic leukodystrophy. Science 341:1233158. 10.1126/science.1233158 23845948

[B17] Bonhoure N. Praz V. Moir R. D. Willemin G. Mange F. Moret C.. (2020). MAF1 is a chronic repressor of RNA polymerase III transcription in the mouse. Sci. Rep. 10:11956. 10.1038/s41598-020-68665-0 32686713 PMC7371695

[B18] Bonkowsky J. L. Nelson C. Kingston J. L. Filloux F. M. Mundorff M. B. Srivastava R. (2010). The burden of inherited leukodystrophies in children. Neurology 75, 718–725. 10.1212/WNL.0b013e3181eee46b 20660364 PMC2931652

[B19] Boucher A. A. Miller W. Shanley R. Ziegler R. Lund T. Raymond G.. (2015). Long-term outcomes after allogeneic hematopoietic stem cell transplantation for metachromatic leukodystrophy: the largest single-institution cohort report. Orphanet J. Rare Dis. 10:94. 10.1186/s13023-015-0313-y 26245762 PMC4545855

[B20] Boycott K. M. Dyment D. A. Sawyer S. L. Vanstone M. R. Beaulieu C. L. (2014). Identification of genes for childhood heritable diseases. Annu. Rev. Med. 65, 19–31. 10.1146/annurev-med-101712-122108 24422568

[B21] Bradbury A. M. Bagel J. H. Nguyen D. Lykken E. A. Pesayco Salvador J. Jiang X.. (2020). Krabbe disease successfully treated *via* monotherapy of intrathecal gene therapy. J. Clin. Invest. 130, 4906–4920. 10.1172/JCI133953 32773406 PMC7456224

[B22] Brüstle O. Spiro A. C. Karram K. Choudhary K. Okabe S. Mckay R. D. (1997). *In vitro*-generated neural precursors participate in mammalian brain development. Proc. Natl. Acad. Sci. U S A 94, 14809–14814. 10.1073/pnas.94.26.14809 9405695 PMC25119

[B23] Buchet D. Garcia C. Deboux C. Nait-Oumesmar B. Baron-Van Evercooren A. (2011). Human neural progenitors from different foetal forebrain regions remyelinate the adult mouse spinal cord. Brain 134, 1168–1183. 10.1093/brain/awr030 21459827

[B24] Burger C. Gorbatyuk O. S. Velardo M. J. Peden C. S. Williams P. Zolotukhin S.. (2004). Recombinant AAV viral vectors pseudotyped with viral capsids from serotypes 1, 2 and 5 display differential efficiency and cell tropism after delivery to different regions of the central nervous system. Mol. Ther. 10, 302–317. 10.1016/j.ymthe.2004.05.024 15294177

[B25] Cartier N. Hacein-Bey-Abina S. Bartholomae C. C. Bougnères P. Schmidt M. Kalle C. V.. (2012). Lentiviral hematopoietic cell gene therapy for X-linked adrenoleukodystrophy. Methods Enzymol. 507, 187–198. 10.1016/B978-0-12-386509-0.00010-7 22365775

[B26] Cartier N. Hacein-Bey-Abina S. Bartholomae C. C. Veres G. Schmidt M. Kutschera I.. (2009). Hematopoietic stem cell gene therapy with a lentiviral vector in X-linked adrenoleukodystrophy. Science 326, 818–823. 10.1126/science.1171242 19892975

[B28] Cearley C. N. Vandenberghe L. H. Parente M. K. Carnish E. R. Wilson J. M. Wolfe J. H. (2008). Expanded repertoire of AAV vector serotypes mediate unique patterns of transduction in mouse brain. Mol. Ther. 16, 1710–1718. 10.1038/mt.2008.166 18714307 PMC3056207

[B27] Cearley C. N. Wolfe J. H. (2006). Transduction characteristics of adeno-associated virus vectors expressing cap serotypes 7, 8, 9 and Rh10 in the mouse brain. Mol. Ther. 13, 528–537. 10.1016/j.ymthe.2005.11.015 16413228

[B29] Chanoumidou K. Mozafari S. Baron-Van Evercooren A. Kuhlmann T. (2020). Stem cell derived oligodendrocytes to study myelin diseases. Glia 68, 705–720. 10.1002/glia.23733 31633852

[B30] Chen H. Mccarty D. M. Bruce A. T. Suzuki K. Suzuki K. (1998). Gene transfer and expression in oligodendrocytes under the control of myelin basic protein transcriptional control region mediated by adeno-associated virus. Gene Ther. 5, 50–58. 10.1038/sj.gt.3300547 9536264

[B31] Choquet K. Forget D. Meloche E. Dicaire M. J. Bernard G. Vanderver A.. (2019a). Leukodystrophy-associated POLR3A mutations down-regulate the RNA polymerase III transcript and important regulatory RNA BC200. J. Biol. Chem. 294, 7445–7459. 10.1074/jbc.RA118.006271 30898877 PMC6509492

[B32] Choquet K. Pinard M. Yang S. Moir R. D. Poitras C. Dicaire M. J.. (2019b). The leukodystrophy mutation POLR3b R103H causes homozygote mouse embryonic lethality and impairs RNA polymerase III biogenesis. Mol. Brain 12:59. 10.1186/s13041-019-0479-7 31221184 PMC6587292

[B33] Choquet K. Yang S. Moir R. D. Forget D. Lariviere R. Bouchard A.. (2017). Absence of neurological abnormalities in mice homozygous for the POLR3a G672E hypomyelinating leukodystrophy mutation. Mol. Brain 10:13. 10.1186/s13041-017-0294-y 28407788 PMC5391615

[B34] Chouery E. Delague V. Jalkh N. Salem N. Kfoury J. Rodriguez D.. (2011). A whole-genome scan in a large family with leukodystrophy and oligodontia reveals linkage to 10q22. Neurogenetics 12, 73–78. 10.1007/s10048-010-0256-3 20721593

[B35] Cloutier P. Coulombe B. (2010). New insights into the biogenesis of nuclear RNA polymerases? Biochem. Cell Biol. 88, 211–221. 10.1139/o09-173 20453924 PMC4492712

[B36] Cong L. Ran F. A. Cox D. Lin S. Barretto R. Habib N.. (2013). Multiplex genome engineering using CRISPR/Cas systems. Science 339, 819–823. 10.1126/science.1231143 23287718 PMC3795411

[B37] Costello D. J. Eichler A. F. Eichler F. S. (2009). Leukodystrophies: classification, diagnosis and treatment. Neurologist 15, 319–328. 10.1097/NRL.0b013e3181b287c8 19901710

[B38] Cota-Coronado A. Diaz-Martinez N. F. Padilla-Camberos E. Diaz-Martinez N. E. (2019). Editing the central nervous system through CRISPR/Cas9 systems. Front. Mol. Neurosci. 12:110. 10.3389/fnmol.2019.00110 31191241 PMC6546027

[B39] Cristofanilli M. Harris V. K. Zigelbaum A. Goossens A. M. Lu A. Rosenthal H.. (2011). Mesenchymal stem cells enhance the engraftment and myelinating ability of allogeneic oligodendrocyte progenitors in dysmyelinated mice. Stem Cells Dev. 20, 2065–2076. 10.1089/scd.2010.0547 21299379

[B41] Daoud H. Tetreault M. Gibson W. Guerrero K. Cohen A. Gburek-Augustat J.. (2013). Mutations in POLR3A and POLR3B are a major cause of hypomyelinating leukodystrophies with or without dental abnormalities and/or hypogonadotropic hypogonadism. J. Med. Genet. 50, 194–197. 10.1136/jmedgenet-2012-101357 23355746

[B42] De Feo D. Merlini A. Laterza C. Martino G. (2012). Neural stem cell transplantation in central nervous system disorders: from cell replacement to neuroprotection. Curr. Opin. Neurol. 25, 322–333. 10.1097/WCO.0b013e328352ec45 22547103

[B43] Degasperis S. M. Bernard G. Wolf N. I. Miller E. Pohl D. (2020). 4H leukodystrophy: mild clinical phenotype and comorbidity with multiple sclerosis. Neurol. Genet. 6:e409. 10.1212/NXG.0000000000000409 32337336 PMC7164972

[B44] Dieci G. Conti A. Pagano A. Carnevali D. (2013). Identification of RNA polymerase III-transcribed genes in eukaryotic genomes. Biochim. Biophys. Acta 1829, 296–305. 10.1016/j.bbagrm.2012.09.010 23041497

[B45] Dieci G. Fiorino G. Castelnuovo M. Teichmann M. Pagano A. (2007). The expanding RNA polymerase III transcriptome. Trends Genet. 23, 614–622. 10.1016/j.tig.2007.09.001 17977614

[B46] Dietrich R. B. Bradley W. G. Zaragoza E. J. T. Otto R. J. Taira R. K. Wilson G. H.. (1988). MR evaluation of early myelination patterns in normal and developmentally delayed infants. Am. J. Roentgenol. 150, 889–896. 10.2214/ajr.150.4.889 2450448

[B47] Dorboz I. Dumay-Odelot H. Boussaid K. Bouyacoub Y. Barreau P. Samaan S.. (2018). Mutation in *POLR3K* causes hypomyelinating leukodystrophy and abnormal ribosomal RNA regulation. Neurol. Genet. 4:e289. 10.1212/NXG.0000000000000289 30584594 PMC6283457

[B48] Duncan I. D. (2005). Oligodendrocytes and stem cell transplantation: their potential in the treatment of leukoencephalopathies. J. Inherit. Metab. Dis. 28, 357–368. 10.1007/s10545-005-7058-z 15868468

[B49] Duncan I. D. Kondo Y. Zhang S.-C. (2011). The myelin mutants as models to study myelin repair in the leukodystrophies. Neurotherapeutics 8, 607–624. 10.1007/s13311-011-0080-y 21979830 PMC3250297

[B50] Eftekharpour E. Karimi-Abdolrezaee S. Wang J. El Beheiry H. Morshead C. Fehlings M. G. (2007). Myelination of congenitally dysmyelinated spinal cord axons by adult neural precursor cells results in formation of nodes of ranvier and improved axonal conduction. J. Neurosci. 27, 3416–3428. 10.1523/JNEUROSCI.0273-07.2007 17392458 PMC6672112

[B51] Ehrlich M. Mozafari S. Glatza M. Starost L. Velychko S. Hallmann A. L.. (2017). Rapid and efficient generation of oligodendrocytes from human induced pluripotent stem cells using transcription factors. Proc. Natl. Acad. Sci. U S A 114, E2243–E2252. 10.1073/pnas.1614412114 28246330 PMC5358375

[B52] Eichler F. Duncan C. Musolino P. L. Orchard P. J. De Oliveira S. Thrasher A. J.. (2017). Hematopoietic stem-cell gene therapy for cerebral adrenoleukodystrophy. N. Engl. J. Med. 377, 1630–1638. 10.1056/NEJMoa1700554 28976817 PMC5708849

[B53] Elbaz B. Popko B. (2019). Molecular control of oligodendrocyte development. Trends Neurosci. 42, 263–277. 10.1016/j.tins.2019.01.002 30770136 PMC7397568

[B521] Elitt M. S. Barbar L. Shick H. E. Powers B. E. Maeno-Hikichi Y. Madhavan M.. (2020). Suppression of proteolipid protein rescues Pelizaeus-Merzbacher disease. Nature 585, 397–403. 10.1038/s41586-020-2494-3 32610343 PMC7810164

[B54] Emery B. (2010). Regulation of oligodendrocyte differentiation and myelination. Science 330, 779–782. 10.1126/science.1190927 21051629

[B55] Escolar M. L. Poe M. D. Provenzale J. M. Richards K. C. Allison J. Wood S.. (2005). Transplantation of umbilical-cord blood in babies with infantile Krabbe’s disease. N. Engl. J. Med. 352, 2069–2081. 10.1056/NEJMoa042604 15901860

[B57] Foust K. D. Nurre E. Montgomery C. L. Hernandez A. Chan C. M. Kaspar B. K. (2009). Intravascular AAV9 preferentially targets neonatal neurons and adult astrocytes. Nat. Biotechnol. 27, 59–65. 10.1038/nbt.1515 19098898 PMC2895694

[B58] Fox I. J. Daley G. Q. Goldman S. A. Huard J. Kamp T. J. Trucco M. (2014). Stem cell therapy. Use of differentiated pluripotent stem cells as replacement therapy for treating disease. Science 345:1247391. 10.1126/science.1247391 25146295 PMC4329726

[B59] Franklin R. J. Ffrench-Constant C. (2008). Remyelination in the CNS: from biology to therapy. Nat. Rev. Neurosci. 9, 839–855. 10.1038/nrn2480 18931697

[B60] Friedmann T. (2001). Stanfield rogers: insights into virus vectors and failure of an early gene therapy model. Mol. Ther. 4, 285–288. 10.1006/mthe.2001.0454 11592829

[B61] Gao G. Alvira M. R. Somanathan S. Lu Y. Vandenberghe L. H. Rux J. J.. (2003). Adeno-associated viruses undergo substantial evolution in primates during natural infections. Proc. Natl. Acad. Sci. U S A 100, 6081–6086. 10.1073/pnas.0937739100 12716974 PMC156329

[B62] Gao G. Vandenberghe L. H. Alvira M. R. Lu Y. Calcedo R. Zhou X.. (2004). Clades of adeno-associated viruses are widely disseminated in human tissues. J. Virol. 78, 6381–6388. 10.1128/JVI.78.12.6381-6388.2004 15163731 PMC416542

[B63] Gao G.-P. Alvira M. R. Wang L. Calcedo R. Johnston J. Wilson J. M. (2002). Novel adeno-associated viruses from rhesus monkeys as vectors for human gene therapy. Proc. Natl. Acad. Sci. U S A 99, 11854–11859. 10.1073/pnas.182412299 12192090 PMC129358

[B64] Garbern J. Y. (2007). Pelizaeus-Merzbacher disease: genetic and cellular pathogenesis. Cell. Mol. Life Sci. 64, 50–65. 10.1007/s00018-006-6182-8 17115121 PMC11136140

[B65] Gauquelin L. Cayami F. K. Sztriha L. Yoon G. Tran L. T. Guerrero K.. (2019). Clinical spectrum of POLR3-related leukodystrophy caused by biallelic POLR1C pathogenic variants. Neurol. Genet. 5:e369. 10.1212/NXG.0000000000000369 32042905 PMC6927361

[B66] Gessler D. J. Li D. Xu H. Su Q. Sanmiguel J. Tuncer S.. (2017). Redirecting *N*-acetylaspartate metabolism in the central nervous system normalizes myelination and rescues Canavan disease. JCI Insight 2:e90807. 10.1172/jci.insight.90807 28194442 PMC5291725

[B67] Gessler D. J. Tai P. W. L. Li J. Gao G. (2019). Intravenous infusion of AAV for widespread gene delivery to the nervous system. Methods Mol. Biol. 1950, 143–163. 10.1007/978-1-4939-9139-6_8 30783972 PMC7339923

[B68] Giedd J. N. Blumenthal J. Jeffries N. O. Castellanos F. X. Liu H. Zijdenbos A.. (1999). Brain development during childhood and adolescence: a longitudinal MRI study. Nat. Neurosci. 2, 861–863. 10.1038/13158 10491603

[B69] Girbig M. Misiaszek A. D. Vorländer M. K. Lafita A. Grötsch H. Baudin F.. (2020). Cryo-EM structures of human RNA polymerase III in its unbound and transcribing states. BioRxiv [Preprint]. 10.1101/2020.06.29.177642 33558764 PMC7610652

[B70] Goldman S. A. (2011). Progenitor cell-based treatment of the pediatric myelin disorders. Arch. Neurol. 68, 848–856. 10.1001/archneurol.2011.46 21403006 PMC3358919

[B71] Goldman S. A. (2017). Progenitor cell-based treatment of glial disease. Prog. Brain Res. 231, 165–189. 10.1016/bs.pbr.2017.02.010 28554396 PMC5662136

[B72] Gonzalez-Perez O. (2012). Neural stem cells in the adult human brain. Biol. Biomed. Rep. 2, 59–69. Available online at: https://www.ncbi.nlm.nih.gov/pmc/articles/PMC3505091/. 23181200 PMC3505091

[B73] Gordon-Lipkin E. Fatemi A. (2018). Current therapeutic approaches in leukodystrophies: a review. J. Child Neurol. 33, 861–868. 10.1177/0883073818792313 30112967 PMC6698898

[B74] Greene C. S. Voight B. F. (2016). Pathway and network-based strategies to translate genetic discoveries into effective therapies. Hum. Mol. Genet. 25, R94–R98. 10.1093/hmg/ddw160 27340225 PMC5036870

[B75] Groeschel S. Kuhl J.-S. Bley A. E. Kehrer C. Weschke B. Doring M.. (2016). Long-term outcome of allogeneic hematopoietic stem cell transplantation in patients with juvenile metachromatic leukodystrophy compared with nontransplanted control patients. JAMA Neurol. 73, 1133–1140. 10.1001/jamaneurol.2016.2067 27400410

[B76] Gruenenfelder F. I. Mclaughlin M. Griffiths I. R. Garbern J. Thomson G. Kuzman P.. (2020). Neural stem cells restore myelin in a demyelinating model of Pelizaeus-Merzbacher disease. Brain 143, 1383–1399. 10.1093/brain/awaa080 32419025 PMC7462093

[B77] Gupta N. Henry R. G. Kang S.-M. Strober J. Lim D. A. Ryan T.. (2019). Long-term safety, immunologic response and imaging outcomes following neural stem cell transplantation for Pelizaeus-Merzbacher disease. Stem Cell Reports 13, 254–261. 10.1016/j.stemcr.2019.07.002 31378671 PMC6700500

[B78] Gupta N. Henry R. G. Strober J. Kang S.-M. Lim D. A. Bucci M.. (2012). Neural stem cell engraftment and myelination in the human brain. Sci. Transl. Med. 4:155ra137. 10.1126/scitranslmed.3004373 23052294 PMC3893824

[B79] Gutierrez M. Thiffault I. Guerrero K. Martos-Moreno G. Á. Tran L. T. Benko W.. (2015). Large exonic deletions in POLR3B gene cause POLR3-related leukodystrophy. Orphanet J. Rare Dis. 10:69. 10.1186/s13023-015-0279-9 26045207 PMC4520020

[B80] Harting I. Al-Saady M. Krageloh-Mann I. Bley A. Hempel M. Bierhals T.. (2020). POLR3A variants with striatal involvement and extrapyramidal movement disorder. Neurogenetics 21, 121–133. 10.1007/s10048-019-00602-4 31940116 PMC7064625

[B81] Helman G. Van Haren K. Bonkowsky J. L. Bernard G. Pizzino A. Braverman N.. (2015). Disease specific therapies in leukodystrophies and leukoencephalopathies. Mol. Genet. Metab. 114, 527–536. 10.1016/j.ymgme.2015.01.014 25684057 PMC4390468

[B82] Hiraide T. Kubota K. Kono Y. Watanabe S. Matsubayashi T. Nakashima M.. (2020a). POLR3A variants in striatal involvement without diffuse hypomyelination. Brain Dev. 42, 363–368. 10.1016/j.braindev.2019.12.012 31932101

[B83] Hiraide T. Nakashima M. Ikeda T. Tanaka D. Osaka H. Saitsu H. (2020b). Identification of a deep intronic POLR3A variant causing inclusion of a pseudoexon derived from an alu element in Pol III-related leukodystrophy. J. Hum. Genet. 65, 921–925. 10.1038/s10038-020-0786-y 32483275

[B84] Hofman M. A. (2014). Evolution of the human brain: when bigger is better. Front. Neuroanat. 8:15. 10.3389/fnana.2014.00015 24723857 PMC3973910

[B85] Horvath P. Barrangou R. (2010). CRISPR/Cas, the immune system of bacteria and archaea. Science 327, 167–170. 10.1126/science.1179555 20056882

[B86] Hsu H. L. Brown A. Loveland A. B. Lotun A. Xu M. Luo L.. (2020). Structural characterization of a novel human adeno-associated virus capsid with neurotropic properties. Nat. Commun. 11:3279. 10.1038/s41467-020-17047-1 32606306 PMC7327033

[B87] Izrael M. Zhang P. Kaufman R. Shinder V. Ella R. Amit M.. (2007). Human oligodendrocytes derived from embryonic stem cells: effect of noggin on phenotypic differentiation *in vitro* and on myelination *in vivo*. Mol. Cell. Neurosci. 34, 310–323. 10.1016/j.mcn.2006.11.008 17196394

[B88] Janson C. Mcphee S. Bilaniuk L. Haselgrove J. Testaiuti M. Freese A.. (2002). Clinical protocol. Gene therapy of Canavan disease: AAV-2 vector for neurosurgical delivery of aspartoacylase gene (ASPA) to the human brain. Hum. Gene. Ther. 13, 1391–1412. 10.1089/104303402760128612 12162821

[B89] Jinek M. East A. Cheng A. Lin S. Ma E. Doudna J. (2013). RNA-programmed genome editing in human cells. eLife 2:e00471. 10.7554/eLife.00471 23386978 PMC3557905

[B90] Johnson S. S. Zhang C. Fromm J. Willis I. M. Johnson D. L. (2007). Mammalian Maf1 is a negative regulator of transcription by all three nuclear RNA polymerases. Mol. Cell 26, 367–379. 10.1016/j.molcel.2007.03.021 17499043

[B91] Jurkiewicz E. Dunin-Wasowicz D. Gieruszczak-Bialek D. Malczyk K. Guerrero K. Gutierrez M.. (2017). Recessive mutations in POLR3B encoding RNA polymerase III subunit causing diffuse hypomyelination in patients with 4H leukodystrophy with polymicrogyria and cataracts. Clin. Neuroradiol. 27, 213–220. 10.1007/s00062-015-0472-1 26478204 PMC5487884

[B92] Kolli N. Lu M. Maiti P. Rossignol J. Dunbar G. L. (2017). CRISPR-Cas9 mediated gene-silencing of the mutant huntingtin gene in an *in vitro* model of Huntington’s disease. Int. J. Mol. Sci. 18:754. 10.3390/ijms18040754 28368337 PMC5412339

[B93] Koob M. Rousseau F. Laugel V. Meyer N. Armspach J. P. Girard N.. (2016). Cockayne syndrome: a diffusion tensor imaging and volumetric study. Br. J. Radiol. 89:20151033. 10.1259/bjr.20151033 27643390 PMC5124826

[B94] Kowalski P. S. Rudra A. Miao L. Anderson D. G. (2019). Delivering the messenger: advances in technologies for therapeutic mRNA delivery. Mol. Ther. 27, 710–728. 10.1016/j.ymthe.2019.02.012 30846391 PMC6453548

[B95] Krivit W. (2004). Allogeneic stem cell transplantation for the treatment of lysosomal and peroxisomal metabolic diseases. Springer Semin. Immunopathol. 26, 119–132. 10.1007/s00281-004-0166-2 15452666

[B96] Krivit W. Peters C. Shapiro E. G. (1999). Bone marrow transplantation as effective treatment of central nervous system disease in globoid cell leukodystrophy, metachromatic leukodystrophy, adrenoleukodystrophy, mannosidosis, fucosidosis, aspartylglucosaminuria, hurler, maroteaux-lamy and sly syndromes and gaucher disease type III. Curr. Opin. Neurol. 12, 167–176. 10.1097/00019052-199904000-00007 10226749

[B97] Krivit W. Sung J. H. Shapiro E. G. Lockman L. A. (1995). Microglia: the effector cell for reconstitution of the central nervous system following bone marrow transplantation for lysosomal and peroxisomal storage diseases. Cell Transplant. 4, 385–392. 10.1016/0963-6897(95)00021-o 7582569

[B98] La Piana R. Cayami F. K. Tran L. T. Guerrero K. Van Spaendonk R. Ounap K.. (2016). Diffuse hypomyelination is not obligate for POLR3-related disorders. Neurology 86, 1622–1626. 10.1212/WNL.0000000000002612 27029625 PMC4844237

[B99] La Piana R. Tonduti D. Gordish Dressman H. Schmidt J. L. Murnick J. Brais B.. (2014). Brain magnetic resonance imaging (MRI) pattern recognition in pol III-related leukodystrophies. J. Child Neurol. 29, 214–220. 10.1177/0883073813503902 24105487

[B100] Laule C. Vavasour I. M. Shahinfard E. Mädler B. Zhang J. Li D. K. B.. (2018). Hematopoietic stem cell transplantation in late-onset krabbe disease: no evidence of worsening demyelination and axonal loss 4 years post-allograft. J. Neuroimaging 28, 252–255. 10.1111/jon.12502 29479774

[B101] Lawlor P. A. Bland R. J. Mouravlev A. Young D. During M. J. (2009). Efficient gene delivery and selective transduction of glial cells in the mammalian brain by AAV serotypes isolated from nonhuman primates. Mol. Ther. 17, 1692–1702. 10.1038/mt.2009.170 19638961 PMC2835020

[B102] Lee Y. Morrison B. M. Li Y. Lengacher S. Farah M. H. Hoffman P. N.. (2012). Oligodendroglia metabolically support axons and contribute to neurodegeneration. Nature 487, 443–448. 10.1038/nature11314 22801498 PMC3408792

[B103] Leone P. Shera D. Mcphee S. W. J. Francis J. S. Kolodny E. H. Bilaniuk L. T.. (2012). Long-term follow-up after gene therapy for canavan disease. Sci. Transl. Med. 4:165ra163. 10.1126/scitranslmed.3003454 23253610 PMC3794457

[B104] Lesniewska E. Boguta M. (2017). Novel layers of RNA polymerase III control affecting tRNA gene transcription in eukaryotes. Open Biol. 7:170001. 10.1098/rsob.170001 28228471 PMC5356446

[B105] Li B. Niu Y. Ji W. Dong Y. (2020). Strategies for the CRISPR-based therapeutics. Trends Pharmacol. Sci. 41, 55–65. 10.1016/j.tips.2019.11.006 31862124 PMC10082448

[B106] Long C. Mcanally J. R. Shelton J. M. Mireault A. A. Bassel-Duby R. Olson E. N. (2014). Prevention of muscular dystrophy in mice by CRISPR/Cas9-mediated editing of germline DNA. Science 345, 1184–1188. 10.1126/science.1254445 25123483 PMC4398027

[B107] Low H. P. Gréco B. Tanahashi Y. Gallant J. Jones S. N. Billings-Gagliardi S.. (2009). Embryonic stem cell rescue of tremor and ataxia in myelin-deficient shiverer mice. J. Neurol. Sci. 276, 133–137. 10.1016/j.jns.2008.09.037 18996543 PMC2650849

[B108] Maeder M. L. Stefanidakis M. Wilson C. J. Baral R. Barrera L. A. Bounoutas G. S.. (2019). Development of a gene-editing approach to restore vision loss in leber congenital amaurosis type 10. Nat. Med. 25, 229–233. 10.1038/s41591-018-0327-9 30664785

[B109] Mahmood A. Raymond G. V. Dubey P. Peters C. Moser H. W. (2007). Survival analysis of haematopoietic cell transplantation for childhood cerebral X-linked adrenoleukodystrophy: a comparison study. Lancet Neurol. 6, 687–692. 10.1016/S1474-4422(07)70177-1 17618834

[B110] Makarova K. S. Koonin E. V. (2015). Annotation and classification of CRISPR-Cas systems. Methods Mol. Biol. 1311, 47–75. 10.1007/978-1-4939-2687-9_4 25981466 PMC5901762

[B111] Mali P. Yang L. Esvelt K. M. Aach J. Guell M. Dicarlo J. E.. (2013). RNA-guided human genome engineering *via* Cas9. Science 339, 823–826. 10.1126/science.1232033 23287722 PMC3712628

[B112] Mariani J. N. Zou L. Goldman S. A. (2019). Human glial chimeric mice to define the role of glial pathology in human disease. Methods Mol. Biol. 1936, 311–331. 10.1007/978-1-4939-9072-6_18 30820907 PMC6700730

[B113] Marteyn A. Sarrazin N. Yan J. Bachelin C. Deboux C. Santin M. D.. (2016). Modulation of the innate immune response by human neural precursors prevails over oligodendrocyte progenitor remyelination to rescue a severe model of Pelizaeus-Merzbacher disease. Stem Cells 34, 984–996. 10.1002/stem.2263 26676415

[B114] Martin H. R. Poe M. D. Provenzale J. M. Kurtzberg J. Mendizabal A. Escolar M. L. (2013). Neurodevelopmental outcomes of umbilical cord blood transplantation in metachromatic leukodystrophy. Biol. Blood Marrow Transplant. 19, 616–624. 10.1016/j.bbmt.2013.01.010 23348427

[B115] Matalon R. Surendran S. Rady P. L. Quast M. J. Campbell G. A. Matalon K. M.. (2003). Adeno-associated virus-mediated aspartoacylase gene transfer to the brain of knockout mouse for canavan disease. Mol. Ther. 7, 580–587. 10.1016/s1525-0016(03)00066-2 12718900

[B116] Matsukawa T. Yamamoto T. Honda A. Toya T. Ishiura H. Mitsui J.. (2020). Clinical efficacy of haematopoietic stem cell transplantation for adult adrenoleukodystrophy. Brain Commun. 2:fcz048. 10.1093/braincomms/fcz048 32954314 PMC7425345

[B117] Mcphee S. W. Francis J. Janson C. G. Serikawa T. Hyland K. Ong E. O.. (2005). Effects of AAV-2-mediated aspartoacylase gene transfer in the tremor rat model of Canavan disease. Mol. Brain Res. 135, 112–121. 10.1016/j.molbrainres.2004.12.007 15857674

[B118] Mendell J. R. Al-Zaidy S. Shell R. Arnold W. D. Rodino-Klapac L. R. Prior T. W.. (2017). Single-dose gene-replacement therapy for spinal muscular atrophy. N. Engl. J. Med. 377, 1713–1722. 10.1056/NEJMoa1706198 29091557

[B119] Mendes M. I. Gutierrez Salazar M. Guerrero K. Thiffault I. Salomons G. S. Gauquelin L.. (2018). Bi-allelic Mutations in EPRS, encoding the glutamyl-prolyl-aminoacyl-tRNA synthetase, cause a hypomyelinating leukodystrophy. Am. J. Hum. Genet. 102, 676–684. 10.1016/j.ajhg.2018.02.011 29576217 PMC5985283

[B120] Meneghini V. Frati G. Sala D. De Cicco S. Luciani M. Cavazzin C.. (2017). Generation of human induced pluripotent stem cell-derived bona fide neural stem cells for *ex vivo* gene therapy of metachromatic leukodystrophy. Stem Cells Transl. Med. 6, 352–368. 10.5966/sctm.2015-0414 28191778 PMC5442804

[B121] Merheb E. Cui M.-H. Dubois J. C. Branch C. A. Gulinello M. Shafit-Zagardo B.. (2020). Defective oligodendrocyte development and function in an RNA polymerase III mutant leukodystrophic mouse. BioRxiv [Preprint]. 10.1101/2020.12.09.418657 34583988 PMC8501794

[B122] Michalski J.-P. Kothary R. (2015). Oligodendrocytes in a Nutshell. Front. Cell. Neurosci. 9:340. 10.3389/fncel.2015.00340 26388730 PMC4556025

[B123] Minnerop M. Kurzwelly D. Wagner H. Soehn A. S. Reichbauer J. Tao F.. (2017). Hypomorphic mutations in POLR3A are a frequent cause of sporadic and recessive spastic ataxia. Brain 140, 1561–1578. 10.1093/brain/awx095 28459997 PMC6402316

[B124] Mitew S. Hay C. M. Peckham H. Xiao J. Koenning M. Emery B. (2014). Mechanisms regulating the development of oligodendrocytes and central nervous system myelin. Neuroscience 276, 29–47. 10.1016/j.neuroscience.2013.11.029 24275321

[B125] Moir R. D. Lavados C. Lee J. Willis I. M. (2020). Functional characterization of Polr3a hypomyelinating leukodystrophy mutations in the *S. cerevisiae* homolog, RPC160. Gene 10.1016/j.gene.2020.145259. [Online ahead of print]. 33148458 PMC8423089

[B126] Monaco M. C. Maric D. Bandeian A. Leibovitch E. Yang W. Major E. O. (2012). Progenitor-derived oligodendrocyte culture system from human fetal brain. J. Vis. Exp.. 10.3791/4274 23288248 PMC3576417

[B127] Monteys A. M. Ebanks S. A. Keiser M. S. Davidson B. L. (2017). CRISPR/Cas9 editing of the mutant huntingtin allele *in vitro* and *in vivo*. Mol. Ther. 25, 12–23. 10.1016/j.ymthe.2016.11.010 28129107 PMC5363210

[B128] Mothe A. J. Tator C. H. (2008). Transplanted neural stem/progenitor cells generate myelinating oligodendrocytes and Schwann cells in spinal cord demyelination and dysmyelination. Exp. Neurol. 213, 176–190. 10.1016/j.expneurol.2008.05.024 18586031

[B129] Musolino P. L. Lund T. C. Pan J. Escolar M. L. Paker A. M. Duncan C. N.. (2014). Hematopoietic stem cell transplantation in the leukodystrophies: a systematic review of the literature. Neuropediatrics 45, 169–174. 10.1055/s-0033-1364179 24459069 PMC4157669

[B130] Neofytou E. O’brien C. G. Couture L. A. Wu J. C. (2015). Hurdles to clinical translation of human induced pluripotent stem cells. J. Clin. Invest. 125, 2551–2557. 10.1172/JCI80575 26132109 PMC4563685

[B131] Nunes M. C. Roy N. S. Keyoung H. M. Goodman R. R. Mckhann G. II Jiang L.. (2003). Identification and isolation of multipotential neural progenitor cells from the subcortical white matter of the adult human brain. Nat. Med. 9, 439–447. 10.1038/nm837 12627226

[B132] Ognjenović J. Simonović M. (2018). Human aminoacyl-tRNA synthetases in diseases of the nervous system. RNA Biol. 15, 623–634. 10.1080/15476286.2017.1330245 28534666 PMC6103678

[B133] Okano H. Temple S. (2009). Cell types to order: temporal specification of CNS stem cells. Curr. Opin. Neurobiol. 19, 112–119. 10.1016/j.conb.2009.04.003 19427192

[B134] Osorio M. J. Goldman S. A. (2016). Glial progenitor cell-based treatment of the childhood leukodystrophies. Exp. Neurol. 283, 476–488. 10.1016/j.expneurol.2016.05.010 27170209 PMC5340082

[B135] Osterman B. Sylvain M. Chouinard S. Bernard G. (2012). Tremor-ataxia with central hypomyelination (TACH): dystonia as a new clinical feature. Mov. Disord. 27, 1829–1830. 10.1002/mds.25270 23208740 PMC4471618

[B136] Parikh S. Bernard G. Leventer R. J. Van Der Knaap M. S. Van Hove J. Pizzino A.. (2015). A clinical approach to the diagnosis of patients with leukodystrophies and genetic leukoencephelopathies. Mol. Genet. Metab. 114, 501–515. 10.1016/j.ymgme.2014.12.434 25655951 PMC4390485

[B137] Park S. G. Schimmel P. Kim S. (2008). Aminoacyl tRNA synthetases and their connections to disease. Proc. Natl. Acad. Sci. U S A 105, 11043–11049. 10.1073/pnas.0802862105 18682559 PMC2516211

[B138] Pelletier F. Perrier S. Cayami F. K. Mirchi A. Saikali S. Tran L. T.. (2020). Endocrine and growth abnormalities in 4H leukodystrophy caused by variants in *POLR3A*, *POLR3B* and *POLR1C*. J. Clin. Endocrinol. Metab. 10.1210/clinem/dgaa700. [Online ahead of print]. 33005949 PMC7823228

[B139] Perrier S. Gauquelin L. Fallet-Bianco C. Dishop M. K. Michell-Robinson M. A. Tran L. T.. (2020). Expanding the phenotypic and molecular spectrum of RNA polymerase III-related leukodystrophy. Neurol. Genet. 6:e425. 10.1212/NXG.0000000000000425 32582862 PMC7238899

[B140] Peters C. Charnas L. R. Tan Y. Ziegler R. S. Shapiro E. G. Defor T.. (2004). Cerebral X-linked adrenoleukodystrophy: the international hematopoietic cell transplantation experience from 1982 to 1999. Blood 104, 881–888. 10.1182/blood-2003-10-3402 15073029

[B141] Pfeiffer S. E. Warrington A. E. Bansal R. (1993). The oligodendrocyte and its many cellular processes. Trends Cell Biol. 3, 191–197. 10.1016/0962-8924(93)90213-k 14731493

[B142] Piguet F. Sondhi D. Piraud M. Fouquet F. Hackett N. R. Ahouansou O.. (2012). Correction of brain oligodendrocytes by AAVrh.10 intracerebral gene therapy in metachromatic leukodystrophy mice. Hum. Gene. Ther. 23, 903–914. 10.1089/hum.2012.015 22642214 PMC3413898

[B143] Poretti A. Meoded A. Fatemi A. (2016). Diffusion tensor imaging: a biomarker of outcome in Krabbe’s disease. J. Neurosci. Res. 94, 1108–1115. 10.1002/jnr.23769 27638596

[B144] Potic A. Brais B. Choquet K. Schiffmann R. Bernard G. (2012). 4H syndrome with late-onset growth hormone deficiency caused by POLR3A mutations. Arch. Neurol. 69, 920–923. 10.1001/archneurol.2011.1963 22451160

[B145] Pouwels P. J. Vanderver A. Bernard G. Wolf N. I. Dreha-Kulczewksi S. F. Deoni S. C.. (2014). Hypomyelinating leukodystrophies: translational research progress and prospects. Ann. Neurol. 76, 5–19. 10.1002/ana.24194 24916848

[B146] Pouya A. Satarian L. Kiani S. Javan M. Baharvand H. (2011). Human induced pluripotent stem cells differentiation into oligodendrocyte progenitors and transplantation in a rat model of optic chiasm demyelination. PLoS One 6:e27925. 10.1371/journal.pone.0027925 22125639 PMC3220701

[B147] Powell S. K. Khan N. Parker C. L. Samulski R. J. Matsushima G. Gray S. J.. (2016). Characterization of a novel adeno-associated viral vector with preferential oligodendrocyte tropism. Gene Ther. 23, 807–814. 10.1038/gt.2016.62 27628693 PMC5541369

[B148] Priller J. Flugel A. Wehner T. Boentert M. Haas C. A. Prinz M.. (2001). Targeting gene-modified hematopoietic cells to the central nervous system: use of green fluorescent protein uncovers microglial engraftment. Nat. Med. 7, 1356–1361. 10.1038/nm1201-1356 11726978

[B149] Privat A. Jacque C. Bourre J. M. Dupouey P. Baumann N. (1979). Absence of the major dense line in myelin of the mutant mouse "shiverer". Neurosci. Lett. 12, 107–112. 10.1016/0304-3940(79)91489-7 460693

[B150] Qian X. Shen Q. Goderie S. K. He W. Capela A. Davis A. A.. (2000). Timing of CNS cell generation: a programmed sequence of neuron and glial cell production from isolated murine cortical stem cells. Neuron 28, 69–80. 10.1016/s0896-6273(00)00086-6 11086984

[B151] Ramsay E. P. Abascal-Palacios G. Daiß J. L. King H. Gouge J. Pilsl M.. (2020). Structure of human RNA polymerase III. Nat. Commun. 11:6409. 10.1038/s41467-020-20262-5 33335104 PMC7747717

[B152] Ran F. A. Hsu P. D. Wright J. Agarwala V. Scott D. A. Zhang F. (2013). Genome engineering using the CRISPR-Cas9 system. Nat. Protoc. 8, 2281–2308. 10.1038/nprot.2013.143 24157548 PMC3969860

[B153] Reina J. H. Azzouz T. N. Hernandez N. (2006). Maf1, a new player in the regulation of human RNA polymerase III transcription. PLoS One 1:e134. 10.1371/journal.pone.0000134 17205138 PMC1762419

[B154] Ricca A. Rufo N. Ungari S. Morena F. Martino S. Kulik W.. (2015). Combined gene/cell therapies provide long-term and pervasive rescue of multiple pathological symptoms in a murine model of globoid cell leukodystrophy. Hum. Mol. Genet. 24, 3372–3389. 10.1093/hmg/ddv086 25749991 PMC4498152

[B155] Richards M. R. Plummer L. Chan Y. M. Lippincott M. F. Quinton R. Kumanov P.. (2017). Phenotypic spectrum of POLR3B mutations: isolated hypogonadotropic hypogonadism without neurological or dental anomalies. J. Med. Genet. 54, 19–25. 10.1136/jmedgenet-2016-104064 27512013 PMC5189673

[B156] Roach A. Takahashi N. Pravtcheva D. Ruddle F. Hood L. (1985). Chromosomal mapping of mouse myelin basic protein gene and structure and transcription of the partially deleted gene in shiverer mutant mice. Cell 42, 149–155. 10.1016/s0092-8674(85)80110-0 2410137

[B157] Rodríguez-Rodríguez D. R. Ramírez-Solís R. Garza-Elizondo M. A. Garza-Rodríguez M. L. Barrera-Saldaña H. A. (2019). Genome editing: a perspective on the application of CRISPR/Cas9 to study human diseases (review). Int. J. Mol. Med. 43, 1559–1574. 10.3892/ijmm.2019.4112 30816503 PMC6414166

[B158] Rogers S. (1959). Induction of arginase in rabbit epithelium by the shope rabbit papilloma virus. Nature 183, 1815–1816. 10.1038/1831815b0 14438381

[B159] Rogers S. (1966). Shope papilloma virus: a passenger in man and its significance to the potential control of the host genome. Nature 212, 1220–1222. 10.1038/2121220a0 21090446

[B160] Rogers S. (1971). Change in the structure of shope papilloma virus-induced arginase associated with mutation of the virus. J. Exp. Med. 134, 1442–1452. 10.1084/jem.134.6.1442 4331301 PMC2139101

[B161] Rogers S. Pfuderer P. (1968). Use of viruses as carriers of added genetic information. Nature 219, 749–751. 10.1038/219749a0 5667075

[B162] Rosenblum D. Gutkin A. Dammes N. Peer D. (2020). Progress and challenges towards CRISPR/Cas clinical translation. Adv. Drug Deliv. Rev. 154-155, 176–186. 10.1016/j.addr.2020.07.004 32659256

[B163] Roth A. D. Ivanova A. Colman D. R. (2006). New observations on the compact myelin proteome. Neuron Glia Biol. 2, 15–21. 10.1017/S1740925X06000068 18634588

[B164] Saitsu H. Osaka H. Sasaki M. Takanashi J.-I. Hamada K. Yamashita A.. (2011). Mutations in POLR3A and POLR3B encoding RNA polymerase III subunits cause an autosomal-recessive hypomyelinating leukoencephalopathy. Am. J. Hum. Genet. 89, 644–651. 10.1016/j.ajhg.2011.10.003 22036171 PMC3213392

[B165] Sampaio-Baptista C. Johansen-Berg H. (2017). White matter plasticity in the adult brain. Neuron 96, 1239–1251. 10.1016/j.neuron.2017.11.026 29268094 PMC5766826

[B166] San Sebastian W. Samaranch L. Heller G. Kells A. P. Bringas J. Pivirotto P.. (2013). Adeno-associated virus type 6 is retrogradely transported in the non-human primate brain. Gene Ther. 20, 1178–1183. 10.1038/gt.2013.48 24067867 PMC3855617

[B167] Sandoval A. Jr. Elahi H. Ploski J. E. (2020). Genetically engineering the nervous system with CRISPR-cas. eNeuro 7:ENEURO.0419-19.2020. 10.1523/ENEURO.0419-19.2020 32098761 PMC7096538

[B168] Sarret C. Lemaire J.-J. Sontheimer A. Coste J. Savy N. Pereira B.. (2018). Brain diffusion imaging and tractography to distinguish clinical severity of human PLP1-related disorders. Dev. Neurosci. 40, 301–311. 10.1159/000492218 30261498

[B169] Sasaki M. Takanashi J.-I. Tada H. Sakuma H. Furushima W. Sato N. (2009). Diffuse cerebral hypomyelination with cerebellar atrophy and hypoplasia of the corpus callosum. Brain Dev. 31, 582–587. 10.1016/j.braindev.2008.09.003 18851904

[B170] Schiffmann R. Van Der Knaap M. S. (2009). Invited article: an MRI-based approach to the diagnosis of white matter disorders. Neurology 72, 750–759. 10.1212/01.wnl.0000343049.00540.c8 19237705 PMC2677542

[B171] Schmidt J. L. Pizzino A. Nicholl J. Foley A. Wang Y. Rosenfeld J. A.. (2020). Estimating the relative frequency of leukodystrophies and recommendations for carrier screening in the era of next-generation sequencing. Am. J. Med. Genet. A 182, 1906–1912. 10.1002/ajmg.a.61641 32573057 PMC11348680

[B172] Schoenemann P. T. Sheehan M. J. Glotzer L. D. (2005). Prefrontal white matter volume is disproportionately larger in humans than in other primates. Nat. Neurosci. 8, 242–252. 10.1038/nn1394 15665874

[B173] Sessa M. Lorioli L. Fumagalli F. Acquati S. Redaelli D. Baldoli C.. (2016). Lentiviral haemopoietic stem-cell gene therapy in early-onset metachromatic leukodystrophy: an *ad hoc* analysis of a non-randomised, open-label, phase 1/2 trial. Lancet 388, 476–487. 10.1016/S0140-6736(16)30374-9 27289174

[B174] Setten R. L. Rossi J. J. Han S.-P. (2019). The current state and future directions of RNAi-based therapeutics. Nat. Rev. Drug Discov. 18, 421–446. 10.1038/s41573-019-0017-4 30846871

[B175] Sevin C. Benraiss A. Van Dam D. Bonnin D. Nagels G. Verot L.. (2006). Intracerebral adeno-associated virus-mediated gene transfer in rapidly progressive forms of metachromatic leukodystrophy. Hum. Mol. Genet. 15, 53–64. 10.1093/hmg/ddi425 16311251

[B176] Sevin C. Verot L. Benraiss A. Van Dam D. Bonnin D. Nagels G.. (2007). Partial cure of established disease in an animal model of metachromatic leukodystrophy after intracerebral adeno-associated virus-mediated gene transfer. Gene Ther. 14, 405–414. 10.1038/sj.gt.3302883 17093507

[B177] Shin J. W. Kim K.-H. Chao M. J. Atwal R. S. Gillis T. Macdonald M. E.. (2016). Permanent inactivation of Huntington’s disease mutation by personalized allele-specific CRISPR/Cas9. Hum. Mol. Genet. 25, 4566–4576. 10.1093/hmg/ddw286 28172889 PMC6078600

[B178] Sim F. J. Mcclain C. R. Schanz S. J. Protack T. L. Windrem M. S. Goldman S. A. (2011). CD140a identifies a population of highly myelinogenic, migration-competent and efficiently engrafting human oligodendrocyte progenitor cells. Nat. Biotechnol. 29, 934–941. 10.1038/nbt.1972 21947029 PMC3365580

[B179] Srivastava S. Cohen J. S. Vernon H. Baranano K. Mcclellan R. Jamal L.. (2014). Clinical whole exome sequencing in child neurology practice. Ann. Neurol. 76, 473–483. 10.1002/ana.24251 25131622

[B180] Steenweg M. E. Vanderver A. Blaser S. Bizzi A. De Koning T. J. Mancini G. M.. (2010). Magnetic resonance imaging pattern recognition in hypomyelinating disorders. Brain 133, 2971–2982. 10.1093/brain/awq257 20881161 PMC3589901

[B181] Sun J. M. Kurtzberg J. (2018). Cell therapy for diverse central nervous system disorders: inherited metabolic diseases and autism. Pediatr. Res. 83, 364–371. 10.1038/pr.2017.254 28985203

[B182] Taft R. J. Vanderver A. Leventer R. J. Damiani S. A. Simons C. Grimmond S. M.. (2013). Mutations in DARS cause hypomyelination with brain stem and spinal cord involvement and leg spasticity. Am. J. Hum. Genet. 92, 774–780. 10.1016/j.ajhg.2013.04.006 23643384 PMC3644624

[B184] Takahashi K. Tanabe K. Ohnuki M. Narita M. Ichisaka T. Tomoda K.. (2007). Induction of pluripotent stem cells from adult human fibroblasts by defined factors. Cell 131, 861–872. 10.1016/j.cell.2007.11.019 18035408

[B183] Takahashi K. Yamanaka S. (2006). Induction of pluripotent stem cells from mouse embryonic and adult fibroblast cultures by defined factors. Cell 126, 663–676. 10.1016/j.cell.2006.07.024 16904174

[B185] Takanashi J.-I. Osaka H. Saitsu H. Sasaki M. Mori H. Shibayama H.. (2014). Different patterns of cerebellar abnormality and hypomyelination between POLR3A and POLR3B mutations. Brain Dev. 36, 259–263. 10.1016/j.braindev.2013.03.006 23643445

[B186] Temple S. (2001). The development of neural stem cells. Nature 414, 112–117. 10.1038/35102174 11689956

[B187] Terao Y. Saitsu H. Segawa M. Kondo Y. Sakamoto K. Matsumoto N.. (2012). Diffuse central hypomyelination presenting as 4H syndrome caused by compound heterozygous mutations in POLR3A encoding the catalytic subunit of polymerase III. J. Neurol. Sci. 320, 102–105. 10.1016/j.jns.2012.07.005 22819058

[B188] Terheggen H. G. Lowenthal A. Lavinha F. Colombo J. P. Rogers S. (1975). Unsuccessful trial of gene replacement in arginase deficiency. Z. Kinderheilkd. 119, 1–3. 10.1007/BF00464689 164740

[B189] Tétreault M. Choquet K. Orcesi S. Tonduti D. Balottin U. Teichmann M.. (2011). Recessive mutations in POLR3B, encoding the second largest subunit of pol III, cause a rare hypomyelinating leukodystrophy. Am. J. Hum. Genet. 89, 652–655. 10.1016/j.ajhg.2011.10.006 22036172 PMC3213403

[B190] Tetreault M. Putorti M. L. Thiffault I. Sylvain M. Venderver A. Schiffmann R.. (2012). TACH leukodystrophy: locus refinement to chromosome 10q22.3-23.1. Can. J. Neurol. Sci. 39, 122–123. 10.1017/s0317167100022174 22384513

[B191] Thiffault I. Wolf N. I. Forget D. Guerrero K. Tran L. T. Choquet K.. (2015). Recessive mutations in POLR1C cause a leukodystrophy by impairing biogenesis of RNA polymerase III. Nat. Commun. 6:7623. 10.1038/ncomms8623 26151409 PMC4506509

[B192] Uchida N. Chen K. Dohse M. Hansen K. D. Dean J. Buser J. R.. (2012). Human neural stem cells induce functional myelination in mice with severe dysmyelination. Sci. Transl. Med. 4:155ra136. 10.1126/scitranslmed.3004371 23052293 PMC3864816

[B193] Uddin F. Rudin C. M. Sen T. (2020). CRISPR gene therapy: applications, limitations and implications for the future. Front. Oncol. 10:1387. 10.3389/fonc.2020.01387 32850447 PMC7427626

[B194] Van Den Broek B. T. A. Page K. Paviglianiti A. Hol J. Allewelt H. Volt F.. (2018). Early and late outcomes after cord blood transplantation for pediatric patients with inherited leukodystrophies. Blood Adv. 2, 49–60. 10.1182/bloodadvances.2017010645 29344584 PMC5761624

[B195] Van Der Knaap M. S. Bugiani M. (2017). Leukodystrophies: a proposed classification system based on pathological changes and pathogenetic mechanisms. Acta Neuropathol. 134, 351–382. 10.1007/s00401-017-1739-1 28638987 PMC5563342

[B196] Van Der Knaap M. S. Valk J. (2005). Magnetic Resonance Imaging of Myelination and Myelin Disorders.. Berlin, Heidelberg: Springer.

[B197] Van Rappard D. F. Konigs M. Steenweg M. E. Boelens J. J. Oosterlaan J. Van Der Knaap M. S.. (2018). Diffusion tensor imaging in metachromatic leukodystrophy. J. Neurol. 265, 659–668. 10.1007/s00415-018-8765-3 29383515 PMC5834549

[B198] Vanderver A. Prust M. Tonduti D. Mochel F. Hussey H. M. Helman G.. (2015). Case definition and classification of leukodystrophies and leukoencephalopathies. Mol. Genet. Metab. 114, 494–500. 10.1016/j.ymgme.2015.01.006 25649058 PMC4390457

[B199] Vanderver A. Simons C. Helman G. Crawford J. Wolf N. I. Bernard G.. (2016). Whole exome sequencing in patients with white matter abnormalities. Ann. Neurol. 79, 1031–1037. 10.1002/ana.24650 27159321 PMC5354169

[B200] Vanderver A. Tonduti D. Bernard G. Lai J. Rossi C. Carosso G.. (2013). More than hypomyelination in Pol-III disorder. J. Neuropathol. Exp. Neurol. 72, 67–75. 10.1097/NEN.0b013e31827c99d2 23242285 PMC3797528

[B201] Vorländer M. K. Baudin F. Moir R. D. Wetzel R. Hagen W. J. H. Willis I. M.. (2020). Structural basis for RNA polymerase III transcription repression by Maf1. Nat. Struct. Mol. Biol. 27, 229–232. 10.1038/s41594-020-0383-y 32066962 PMC7104376

[B202] Vrij-Van Den Bos S. Hol J. A. La Piana R. Harting I. Vanderver A. Barkhof F.. (2017). 4H leukodystrophy: a brain magnetic resonance imaging scoring system. Neuropediatrics 48, 152–160. 10.1055/s-0037-1599141 28561206

[B203] Wang S. Bates J. Li X. Schanz S. Chandler-Militello D. Levine C.. (2013). Human iPSC-derived oligodendrocyte progenitor cells can myelinate and rescue a mouse model of congenital hypomyelination. Cell Stem Cell 12, 252–264. 10.1016/j.stem.2012.12.002 23395447 PMC3700553

[B204] White R. J. (2011). Transcription by RNA polymerase III: more complex than we thought. Nat. Rev. Genet. 12, 459–463. 10.1038/nrg3001 21540878

[B205] Willis I. M. Moir R. D. (2018). Signaling to and from the RNA polymerase III transcription and processing machinery. Annu. Rev. Biochem. 87, 75–100. 10.1146/annurev-biochem-062917-012624 29328783 PMC6038698

[B206] Windrem M. S. Nunes M. C. Rashbaum W. K. Schwartz T. H. Goodman R. A. Mckhann G.. (2004). Fetal and adult human oligodendrocyte progenitor cell isolates myelinate the congenitally dysmyelinated brain. Nat. Med. 10, 93–97. 10.1038/nm974 14702638

[B207] Windrem M. S. Schanz S. J. Guo M. Tian G.-F. Washco V. Stanwood N.. (2008). Neonatal chimerization with human glial progenitor cells can both remyelinate and rescue the otherwise lethally hypomyelinated shiverer mouse. Cell Stem Cell 2, 553–565. 10.1016/j.stem.2008.03.020 18522848 PMC3358921

[B208] Windrem M. S. Schanz S. J. Morrow C. Munir J. Chandler-Militello D. Wang S.. (2014). A competitive advantage by neonatally engrafted human glial progenitors yields mice whose brains are chimeric for human glia. J. Neurosci. 34, 16153–16161. 10.1523/JNEUROSCI.1510-14.2014 25429155 PMC4244478

[B209] Windrem M. S. Schanz S. J. Zou L. Chandler-Militello D. Kuypers N. J. Nedergaard M.. (2020). Human glial progenitor cells effectively remyelinate the demyelinated adult brain. Cell Rep. 31:107658. 10.1016/j.celrep.2020.107658 32433967 PMC8237530

[B210] Wirth T. Parker N. Ylä-Herttuala S. (2013). History of gene therapy. Gene 525, 162–169. 10.1016/j.gene.2013.03.137 23618815

[B211] Wolf N. I. Harting I. Innes A. M. Patzer S. Zeitler P. Schneider A.. (2007). Ataxia, delayed dentition and hypomyelination: a novel leukoencephalopathy. Neuropediatrics 38, 64–70. 10.1055/s-2007-985137 17712733

[B212] Wolf N. I. Salomons G. S. Rodenburg R. J. Pouwels P. J. Schieving J. H. Derks T. G.. (2014a). Mutations in RARS cause hypomyelination. Ann. Neurol. 76, 134–139. 10.1002/ana.24167 24777941

[B522] Wolf N. I. ffrench-Constant C. van der Knaap M. S. (2020). Hypomyelinating leukodystrophies - unravelling myelin biology. Nat. Rev. Neurol. 10.1038/s41582-020-00432-1. [Online ahead of print]. 33324001

[B214] Wolff A. Koch M. J. Benzinger S. Van Waes H. Wolf N. I. Boltshauser E.. (2010). Rare dental peculiarities associated with the hypomyelinating leukoencephalopathy 4H syndrome/ADDH. Pediatr. Dent. 32, 386–392. Available online at: https://aapd.publisher.ingentaconnect.com/contentone/aapd/pd/2010/00000032/00000005/art00003#. 21070704

[B213] Wolf N. I. Vanderver A. Van Spaendonk R. M. Schiffmann R. Brais B. Bugiani M.. (2014b). Clinical spectrum of 4H leukodystrophy caused by POLR3A and POLR3B mutations. Neurology 83, 1898–1905. 10.1212/WNL.0000000000001002 25339210 PMC4248461

[B215] Wolpert L. (1994). Positional information and pattern formation in development. Dev. Genet. 15, 485–490. 10.1002/dvg.1020150607 7834908

[B216] Wright M. D. Poe M. D. Derenzo A. Haldal S. Escolar M. L. (2017). Developmental outcomes of cord blood transplantation for Krabbe disease: A 15-year study. Neurology 89, 1365–1372. 10.1212/WNL.0000000000004418 28855403 PMC5649761

[B217] Wu C.-C. Herzog F. Jennebach S. Lin Y.-C. Pai C.-Y. Aebersold R.. (2012). RNA polymerase III subunit architecture and implications for open promoter complex formation. Proc. Natl. Acad. Sci. U S A 109, 19232–19237. 10.1073/pnas.1211665109 23132938 PMC3511066

[B218] Wu S. Bai Z. Dong X. Yang D. Chen H. Hua J.. (2019). Novel mutations of the POLR3A gene caused POLR3-related leukodystrophy in a chinese family: a case report. BMC Pediatr. 19:289. 10.1186/s12887-019-1656-7 31438894 PMC6704677

[B219] Yandava B. D. Billinghurst L. L. Snyder E. Y. (1999). “Global” cell replacement is feasible *via* neural stem cell transplantation: evidence from the dysmyelinated shiverer mouse brain. Proc. Natl. Acad. Sci. U S A 96, 7029–7034. 10.1073/pnas.96.12.7029 10359833 PMC22044

[B220] Yang L. Mali P. Kim-Kiselak C. Church G. (2014). CRISPR-Cas-mediated targeted genome editing in human cells. Methods Mol. Biol. 1114, 245–267. 10.1007/978-1-62703-761-7_16 24557908

[B221] Yang S. Chang R. Yang H. Zhao T. Hong Y. Kong H. E.. (2017). CRISPR/Cas9-mediated gene editing ameliorates neurotoxicity in mouse model of Huntington’s disease. J. Clin. Invest. 127, 2719–2724. 10.1172/JCI92087 28628038 PMC5490741

[B222] Yin H. Xue W. Chen S. Bogorad R. L. Benedetti E. Grompe M.. (2014). Genome editing with Cas9 in adult mice corrects a disease mutation and phenotype. Nat. Biotechnol. 32, 551–553. 10.1038/nbt.2884 24681508 PMC4157757

[B223] Zerah M. Piguet F. Colle M.-A. Raoul S. Deschamps J.-Y. Deniaud J.. (2015). Intracerebral gene therapy using aavrh.10-harsa recombinant vector to treat patients with early-onset forms of metachromatic leukodystrophy: preclinical feasibility and safety assessments in nonhuman primates. Hum. Gene Ther. Clin. Dev. 26, 113–124. 10.1089/humc.2014.139 25758611

[B224] Zhao X. Moore D. L. (2018). Neural stem cells: developmental mechanisms and disease modeling. Cell Tissue Res. 371, 1–6. 10.1007/s00441-017-2738-1 29196810 PMC5963504

